# Insect Diversity in Eastern Mediterranean Maquis Exhibits Extensive Variation Between Sites and Years, Complicating Monitoring Efforts

**DOI:** 10.3390/biology15211945

**Published:** 2026-10-27

**Authors:** Meir Finkel, Ariel Leib Leonid Friedman, Gilad Ben Zvi, Orr Comay

**Affiliations:** The Steinhardt Museum of Natural History, Tel Aviv University, 12 Klausner Street, Tel Aviv 6997801, Israel; laibale@tauex.tau.ac.il (A.L.L.F.); giladbenzvi@tauex.tau.ac.il (G.B.Z.); orrcomay@tauex.tau.ac.il (O.C.)

**Keywords:** long-term monitoring, baseline, community composition, insects, beetles, east mediterranean

## Abstract

Insects are understudied in the Eastern Mediterranean, a region with heterogeneous biotas facing rapid regional warming and land-use changes. It is essential to establish a baseline for insect monitoring, including the extent of spatial, inter-annual, and seasonal variations in insect diversity and composition—all prominent in the Mediterranean. To assess these, we conducted an intensive three-year (2022–2024) sampling of ants and plant-dwelling beetles at three sites along Mt. Meron’s maquis-covered western slopes (Galilee, Israel, at 550, 850, and 1050 m above sea level), with similar tree, shrub, mammal, and bird communities. Results show high inter-annual variability of beetle species (~15% of species were collected in all three years; ~ 65% only in one of the study years), and a lesser but still significant spatial variation between sites (~50% in only one of the sites; 20% in all sites). In both respects, 30 collection events have not reached 90% of the total multi-annual detected species list. In ants, similar but less pronounced results were found. These findings underscore the need for a multi-year, multi-site intensive sampling effort to compile a complete species list as a baseline for trend monitoring of the Mediterranean maquis and warrant a discussion on its methodology.

## Simple Summary

Insects are an important part of natural ecosystems, but they are still poorly studied in the Eastern Mediterranean, where rapid climate warming and changes in land use may affect their diversity. To establish a reliable starting point for future monitoring, we studied beetles and ants living on plants for three years (2022–2024) at three locations at different elevations on the western slopes of Mt. Meron. We wanted to understand how much insect communities change between years and sites. We found that beetle species varied greatly from year to year: only about 15% were found in all three years, while about 65% were recorded in only one year. Differences between locations were also substantial. Even after 30 sampling visits, we had not recorded 90% of all the species found during the entire study. Ants showed similar, though less pronounced, patterns. Our results show that a single year or location is not enough to provide a complete picture of insect diversity. Long-term monitoring should therefore include repeated sampling over several years and at several locations. Such reliable baseline information will help society detect future changes in biodiversity and better understand the effects of climate and environmental change.

## 1. Introduction

The Eastern Mediterranean Basin, and specifically the Levant, is known for its biological heterogeneity compared to neighboring temperate and desert regions [1,2,3,4,5,6]. The factors enabling this diversity stem mainly from the region’s location at the junction of Africa, Asia, and Europe, which creates a species-rich biogeographic unit [7,8]. The Levant includes three primary climate zones: Mediterranean, steppe, and desert, which hold diverse habitats and different phytogeographical and zoogeographical zones. Our research was conducted in northern Israel, a country with a profound south (Negev Desert region)—north (Upper Galilee Mediterranean region) climatic and biogeographical gradient [8]. Israel is also characterized by rapid demographic and economic growth, both of which drive biodiversity declines. For instance, between 2000 and 2023, Israel’s population grew by 57%, with an average yearly growth of 2.0%; Gross Domestic Product (GDP) grew by an annual mean of 4.1% (2.1% per capita) in 2012–2022 [9], while built-up area reached 18.2% of the total area north of Beersheba (i.e., the Mediterranean part of Israel) [10]. Additionally, the Eastern Mediterranean warmed by 0.45° C per decade in 1981–2019, faster than the global trend (0.27° C per decade) and faster than most inhabited regions of the world [11]. Thus, the Israeli case exemplifies the general phenomenon of higher biodiversity sensitivity of Mediterranean habitats to land-use and climate change, compared to most regions of the world [12].

Arthropods are known to rapidly decline throughout the world, making this ultra-diverse group a high priority for nature conservation [13,14,15,16,17,18,19]. Efficient conservation management, whether local or regional, must be based on extensive knowledge of its biodiversity and its spatial and temporal patterns, hand in hand with long-term monitoring to assess the impacts of the aforementioned anthropogenic factors. The Levant, however, suffers from a lack of in-depth arthropod biodiversity research ([20] and references therein) which also hinders long-term arthropod monitoring programs in this region [18,21,22]. In Israel, where the few biodiversity monitoring programs in the region are concentrated, most are focused on vegetation and vertebrates, while the monitoring of arthropods (which constitute, as elsewhere, most of the biodiversity) is in very early stages [10,23,24,25]. Hence, it offers few ecological insights, and detailed ecological information on the local biodiversity is only available from focused studies; however, these may be limited in time and extent. Notably, for assessing long-term trends in biodiversity, researchers need a solid baseline for comparison. Here, we aim to provide a baseline for Eastern Mediterranean arthropods of the Mediterranean maquis using a large, multi-year, multi-site (across an altitudinal gradient) study covering two important and diverse groups: beetles and ants.

## 2. Materials and Methods

### 2.1. Research Sites, Vegetation and Climate

**Figure 1:**
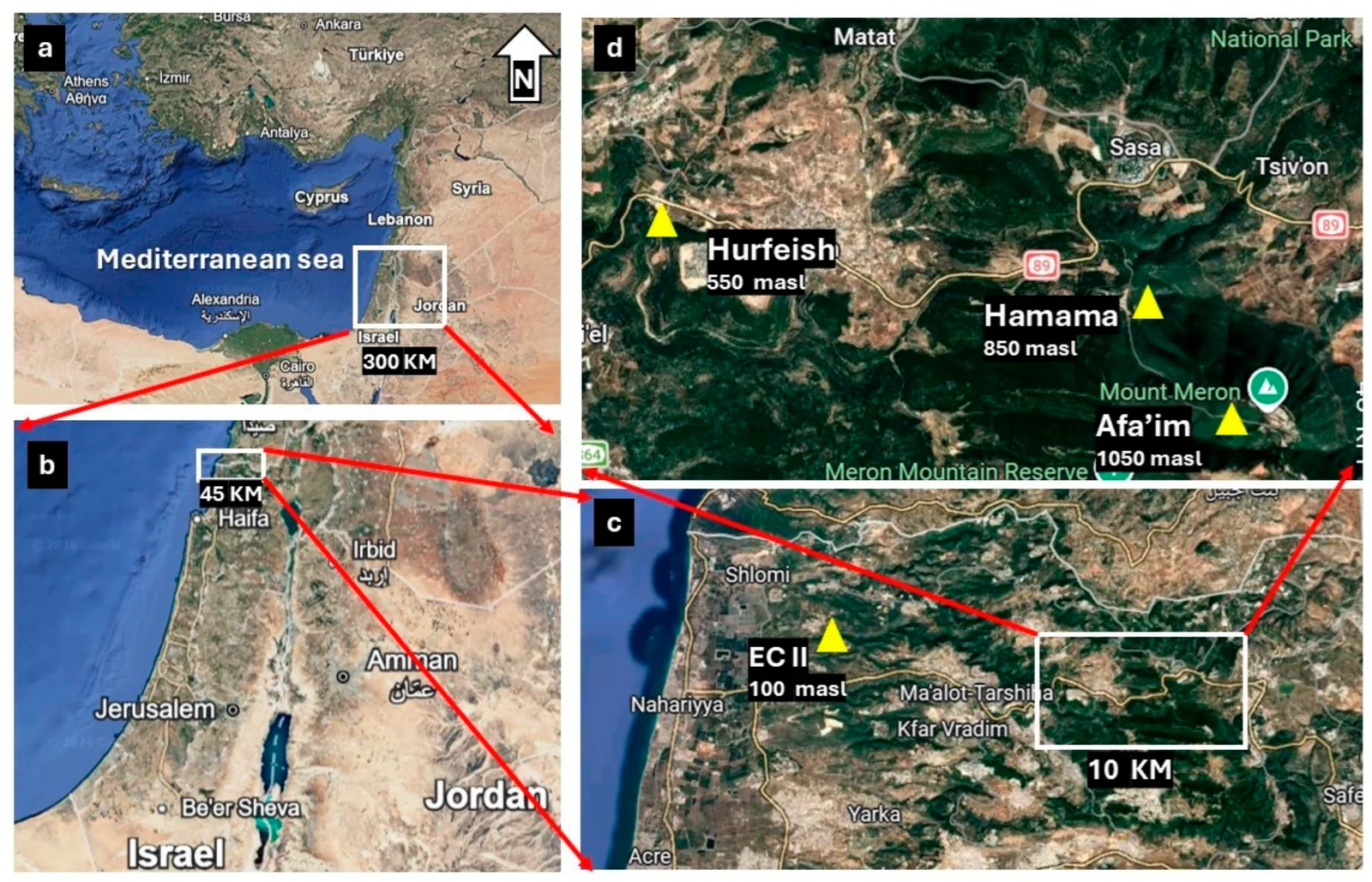
The research region (**a**,**b**), area (**c**), and sites (**d**). Orthophoto from Google. (2026). Google Maps. (https://www.google.com/maps/@33.0324979,35.3195846,34127m/data=!3m1!1e3!5m1!1e1?entry=ttu&g_ep=EgoyMDI2MTAwNy4wIKXMDSoASAFQAw%3D%3D accessed on 10 September 2026).

The research was conducted at three sites within Mt. Meron Nature Reserve (declared in 1965) in the Western Upper Galilee, on the west slope of Mt. Meron (Peak altitude: 1208 m a.s.l): Hurfeish, Hamama and Afa’im (35.3254848, 33.0218292, 550; 35.3949487, 33.0081501, 850, and 35.4119217, 32.9953373, 1050 m a.s.l., respectively; Figure 1). Each site was ~10,000 m^2^ in size. The ground in each of the sites is flat to 10% inclination (east–west). The Galilee region is characterized by relatively homogeneous flora, with the following average coverage of woody plants: trees (49%); shrubs (35%); semi-shrubs (13%); geophytes (1%); and hemicryptophytes (2%). The relative portion of the most common woody species (trees and shrubs) in the Galilee region is: *Quercus coccifera* (33%); *Calicotome villosa* (12%); *Smilax aspera* (11%) [10]. In general, the western slopes of Mt. Meron are densely covered by a *Quercus coccifera* and *Pistacia terebinthus* plant association reaching 4–5 m. in height [26,27]. The sites we selected for our research are composed of the three habitats found in the Mediterranean maquis of the Western Upper Galilee—oak trees, shrub garigue, and open grassland—each around a third of the site area (and collection effort divided accordingly). The sites were not affected by grazing. Based on a vegetation survey conducted in the first year of research, our study sites show homogeneity in the more prominent taxa: trees: mainly *Quercus coccifera* and *Pistacia terebinthus*, *Quercus boissieri Reut.*, *Pyrus syriaca Boiss.* In the Hamama site collection was conducted also from *Prunus ursina Kotschy* trees, which do not grow in the other sites. Shrubs (all flowering) are mainly *Calicotome villosa*, followed in frequency *by Spartium junceum*, *Cistus salvifolius*, *Cistus creticus* and *Sarcopoterium spinosum*. Main arthropod-hosting annual and biennial species: *Daucus carota* subsp. Maximus, *Notobasis syriaca*, *Silybum marianum*, *Centaurea iberica*, and *Cichorium endivia* L.

The shrubs and annuals are similar to those found in Evolution Canyon II—ECII, row 4 (the dominant tree there being *Quercus coccifera* [24]). The Western Upper Galilee is relatively homogeneous in its vertebrate community, with all dominant tree, mammal, bird, and reptile species prevalent throughout the maquis in the mountains and hills of the Levant (i.e., in Israel, Lebanon and Syria) [28,29]. This regional homogeneity and our previous findings regarding long-term (20-year) arthropod species turnover at an adjacent site (EC II; [24]) serve as the background for our current research, which aims to establish a baseline for long-term arthropod monitoring in this region.

The geology and soil at the three sites, as well as at the ECII Hardalit site (~100 m a.s.l; [24]), are similar: limestone deposited in the Cenomanian age [30] and Terra-rossa soil, which develops on limestone. The three sites share similar mean temperature, humidity, and solar radiation (Appendix A: Table A1). While the average annual rainfall in this region is 955 mm, and limited differences between sites exist (Appendix A: Table A1), it is important to note that botanists define this area as the “rainy Galilee” and claim that when annual precipitation exceeds 600 mm, the main factor determining plant composition is rock formation and soil [27], which is shared by the three sites, and that ~ 200 mm of inter-annual differences does not affect changes in vegetation [26]. There were significant yearly differences during 2022–2024 research period (as measured in Harashim meteorological station in Mt. Meron, from 1986: Israel Meteorological Service website: URL: https://data.gov.il/he/datasets/meteorological_service/481 (accessed on 1 April 2026): winter 2021–2022: 925 mm; winter 2022–2023: 683 mm; winter 2023–2024: 1248 mm). However, given that only three years were surveyed, we could not incorporate this variation explicitly into the modeling (see Section 2.4).

### 2.2. Beetle and Ant Collection

**Figure A1:**
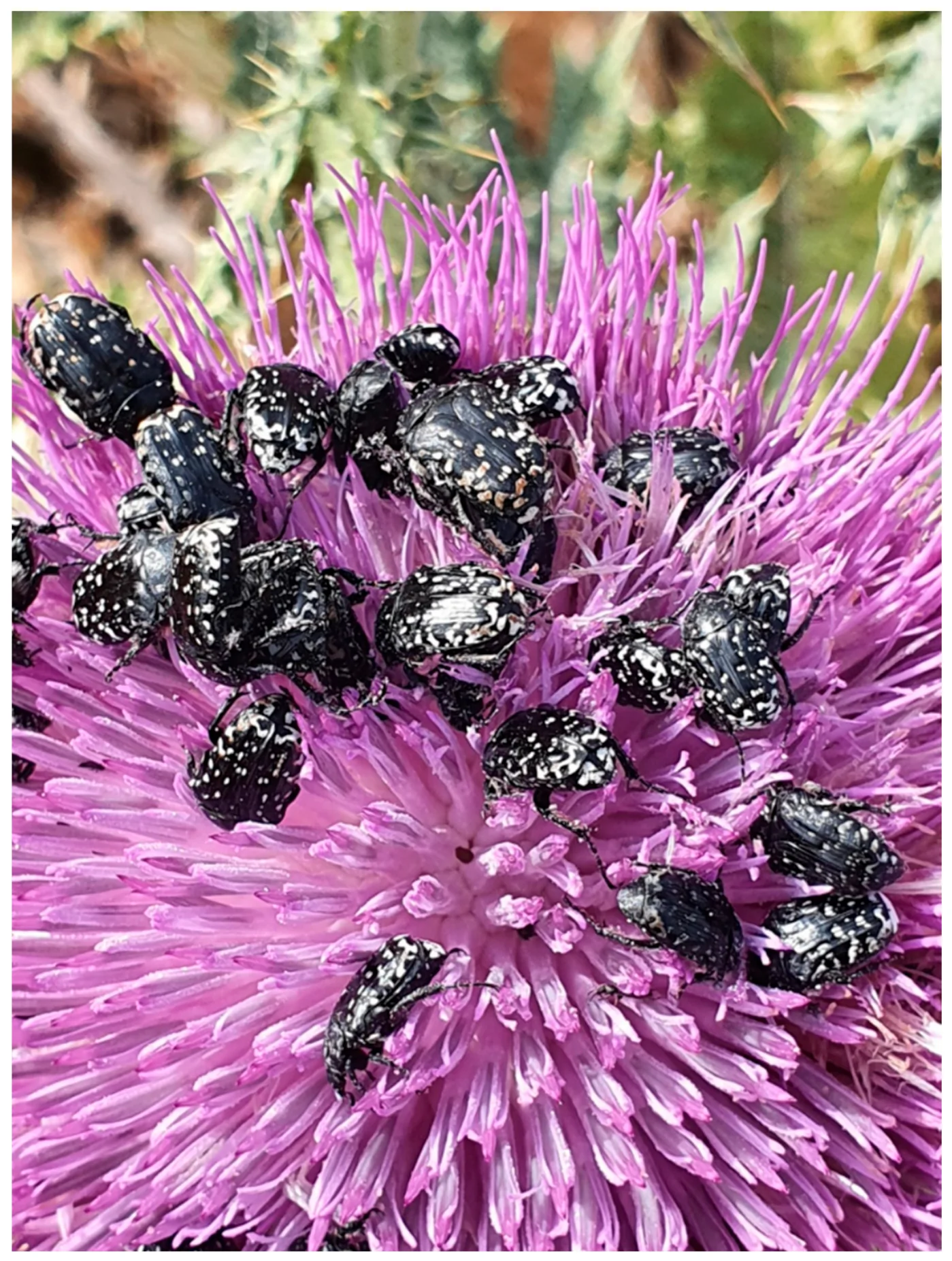
*Onopordum cynarocephalum*, Hurfeish. 28 May 2022.

**Figure A2:**
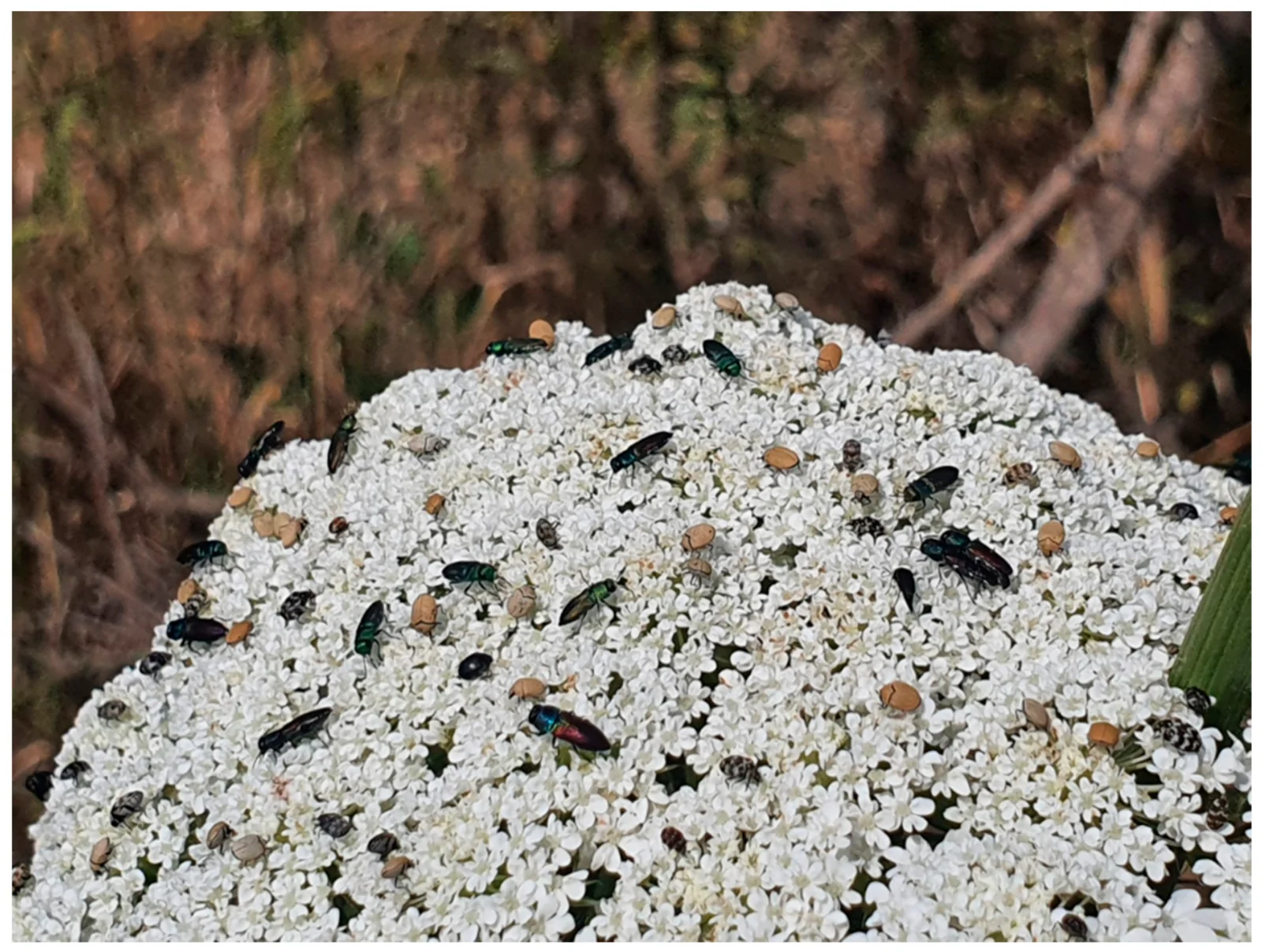
*Daucus carota* subsp. Maximus, Hurfeish, 3 June 2022.

Collection with a sweeping net was conducted during the 2022–2024 spring seasons from late March (end of winter) to early July (when annuals become totally dry), every ~7 days (between 13 and 16 collection events each year, at the three sites, 38–47 per year; 126–131 in three years). The total sweeping duration was 45 min at each site in each collection, conducted by one person for 45 min or by three persons for 15 min, spread evenly between trees, shrubs, and annuals. The method is effective for collecting diurnal flying beetles and arboreal ant species. Ants were also actively collected from the ground (including from nests). Reliable abundance of flying beetles and arboreal ants is very difficult to measure because, during the Mediterranean spring, single flowers can host dozens of specimens (see Appendix A, Figure A1 and Figure A2), making comparisons between collections and sites problematic, as even adding one sweep on a particular flower can make a substantial difference and result in biased data. Additionally, collecting all specimens in each collection would probably result in local extinction even within the same season, therefore hampering the research aims. For that reason, in each collection we caught only 1–2 specimens of a species at each site. Therefore, species collected by sweeping nets were analyzed only as detected or undetected, resulting in a per-visit detected species list. Therefore, the term “species list” herein should be interpreted as “detected species list under applied survey efforts”. While we could not infer and analyze abundance and population trends directly, given our focus on species lists, frequently versus infrequently detected species could be easily identified from the full list of beetles and ants given in Supplementary Materials (Files S9–S16) and also briefly discussed below.

In the inter-site comparison, results of beetle collection were compared to the findings from the 2020–2021 collection in EC II—Row 4—Valley bottom (Hardalit site), with the same floristic characteristics, at the same season and collection time, and using the same methods [24]. Due to the differences in years and climatic variables, we note that the comparison is contextual and descriptive. In ants, we did not compare results to those obtained from ECII, because the collection there also included the use of pitfall traps.

In order to detect specific families which may present substantially higher or lesser rates of inter-annual or inter-site overlap, we also analyzed seven families with more than 19 species: Apionidae; Buprestidae; Cerambycidae; Chrysomelidae; Coccinellidae; Curculionidae and Melyridae.

### 2.3. Species Identification

Beetle identification was conducted by A.L.L.F. All collected specimens are deposited in the Steinhardt Museum of Natural History, Tel Aviv University (SMNHTAU). We focused our identification effort on nine beetles superfamilies/families with the highest number of species (Buprestidae; Cerambycidae; Chrysomelidae (including Bruchinae); Coccinellidae; Curculionoidea (including Anthribidae, Apionidae, Brachyceridae, Curculionidae and Rhynchitidae); Dermestidae; Melyridae; Scarabaeoidea (including Glaphyridae, Ochodaeidae and Scarabaeidae). Ant identification was conducted at SMNHTAU, where all specimens are deposited. In total, 317 beetle species and 50 ant species were identified. All species were checked against the identified material in SMNHTAU. Above 80% of the specimens were identified to the species level; the rest were identified to the genus level and separated into morphospecies.

### 2.4. Statistical Analysis

We studied six aspects of insect biodiversity in the three study sites during the three research years: (1) relative overlap of species lists per year-site combination; (2) species composition using model-based ordinations using copulas on year-site species detection data; (3) species-specific preferences of site, year and season, using Generalized Linear Latent Variable Models (GLLVMs) on date-site species detection data; (4) factors affecting survey-specific (with survey defined as a unique site-date combination) detected species richness, including seasonality, year and site identity, using Generalized Linear Models (GLMs), while testing for temporal autocorrelation; (5) minimum survey effort required to observe at least 90% of the full observed species lists obtained in the whole study per year, per site and per year-site combination, using samples of the possible combinations of surveys when choosing K surveys out of N; (6) We generated species accumulation curves based on rarefaction and extrapolation methods based on visit-specific species lists [31].

In analysis 5 (species lists when choosing K surveys out of N), we examined all possible combinations up to K = 12 for individual year-site combinations. This maximum K of 12 reflects one visit fewer than the lowest number of actual visits in any year-site combination, which was 13 (see Section 2.2). However, the immense number of possible combinations when examining either sites pooled across all years, or years pooled across all sites, made an exhaustive approach computationally unfeasible. Therefore, for these broader analyses, we randomly sampled 5000 combinations instead of exploring all possibilities.

All six aspects were studied separately for ants and for beetles. We conducted the statistical analysis in R version 4.6.1 [32], using several packages including tidyverse version 2.0.0 [33], data.table version 1.18.4 [34], glmmTMB version 1.1.14 [35,36], here version 1.0.2 [37], ggvenn version 0.1.19 [38], iNEXT version 3.0.2 [39], gllvm version 2.0.13 [40] and ecoCopula version 1.0.6 [41]. The full R scripts we used for this analysis are given as Rmarkdown files and are available as Supplementary Materials (Files S3–S8), along with their input data Excel files (Files S1 and S2). In summary, we used the Akaike Information Criterion [42] in a stepwise backward model selection process when fitting GLMs or GLLVMs, where we first started with a full model including all predictors (site identity, year and seasonality, including the interaction between the latter) and gradually removed them. We modeled seasonality based on the cosinor approach, i.e., using the sine and cosine of the radian distance of the survey date from the summer solstice (calculated as π times the number of days before the summer solstice divided by half the number of days in a year) as predictors and allowed for up to two annual peaks and troughs by including also the sine and cosine of double this radian distance [43]. The years were treated as categorical rather than continuous predictors, since we did not aim to detect a long-term trend. Annual weather variables (e.g., precipitation and temperature) were not directly used as continuous predictors due to the low number of year-site combinations (three sites sampled over three years = nine total year-site combinations), and the potential confounding factors related to inter-annual differences besides weather patterns. All reported *p*-values were adjusted for multiple comparisons (between years and sites) based on Benjamini–Hochberg’s False Discovery Rate [44]. For *p*-value adjustment, we grouped together the *p*-values of tests of the same taxon (beetles or ants) and the same analysis (numbered 1–5 above); e.g., predictor *p*-values of the ant species richness per visit model. For the GLLVMs (analysis 3), we adjusted the *p*-values per model, since we fitted the chosen GLLVM twice per taxon: once using Afa’im as the reference site and 2022 as the reference year and once using Hamama as the reference site and 2023 as the reference year. This allowed us to make all pairwise comparisons between the three sites and three years. We used the model with Afa’im as the reference site and 2022 as the reference year for the *p*-value adjustment whenever possible.

## 3. Results

The full species lists (including the number of detections and percent of detections out of the total number of surveys conducted per species) are available as Supplementary Materials (Files S9–S16). The species lists are divided by taxa (beetles and ants), and each is given in four aggregations: whole data (not separated by years or site), aggregated by site, aggregated by year, and aggregated by both year and site. These tables allow an assessment of relative abundance/detectability of the different species.

### 3.1. Beetles

#### 3.1.1. Inter-Annual Differences

Table 1 summarizes the species overlap analysis between years (analysis 1 in Section 2.4). In each of the three sites, ~two-thirds of the species appear in only one of the three years, with the remaining third split between species appearing in 2/3 years and 3/3 years. The percentages are strikingly similar across sites, and this phenomenon persists (slightly moderated) when all three sites are combined.

Analyzing the seven most common families shows differences, with Apionidae as the most “stable” one regarding temporality, and Coccinellidae as the least “stable” one (Table 2).

**Figure 2:**
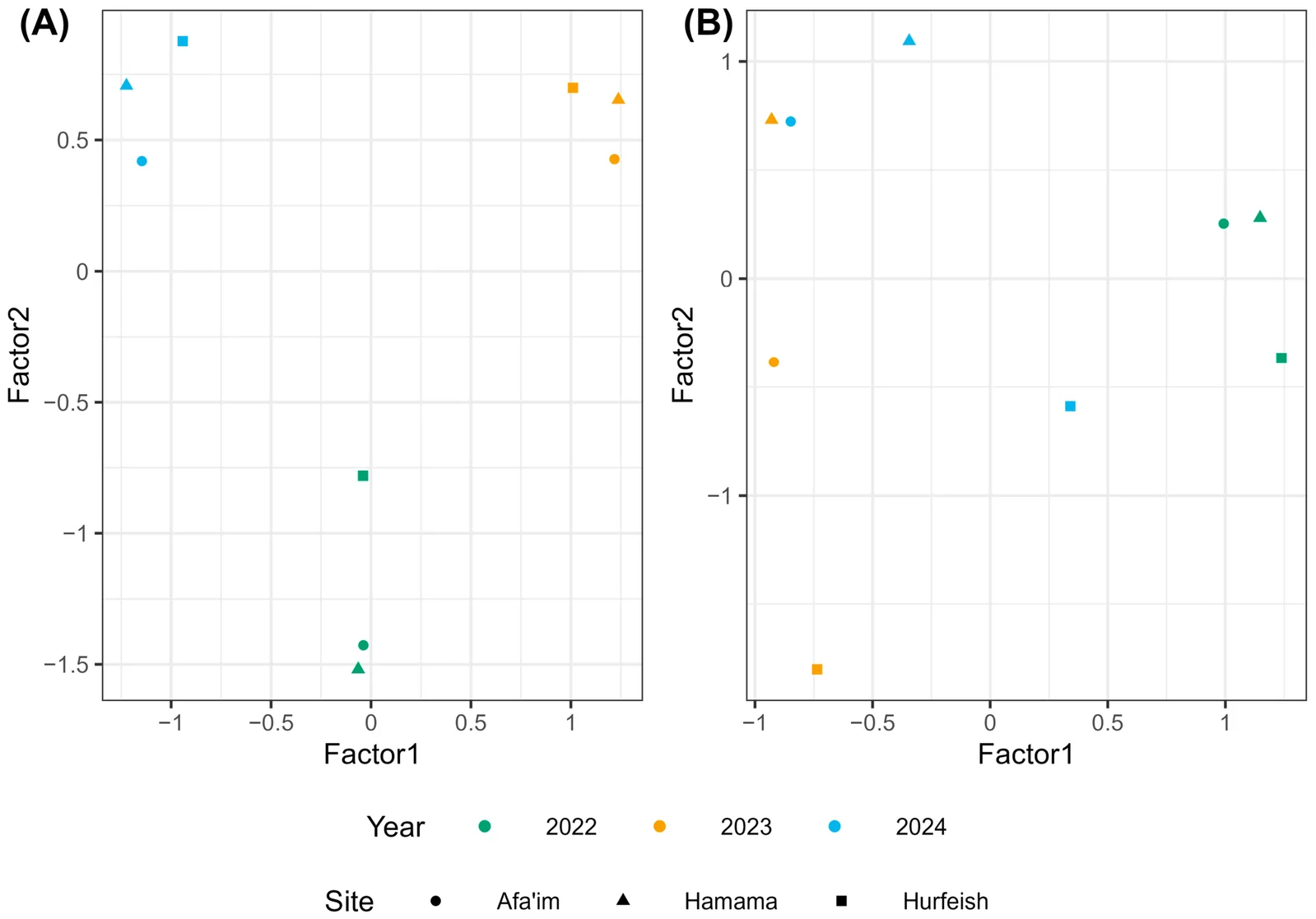
Model-based ordination of insect species detection by year-site combination data. (**A**) Beetles. (**B**) Ants.

Beetle species composition per site-year aggregated by year rather than by site, as demonstrated in the model-based ordination of the site-year species detection data (analysis 2 in Section 2.4; Figure 2A).

The chosen beetle GLLVM for the date-site data (analysis 3 in Section 2.4) found 26 beetle species that were significantly more common in 2023 compared to 2022, while 32 species were significantly more common in 2022 vs. 2023; 21 beetle species were significantly more common in 2024 compared to 2022, while 29 species were significantly more common in 2022 vs. 2024. Twenty-seven beetle species were significantly more common in 2024 compared to 2023, while 30 species were significantly more common in 2023 vs. 2024. The full list of species that significantly differed between years is given in Appendix B.

**Figure 3:**
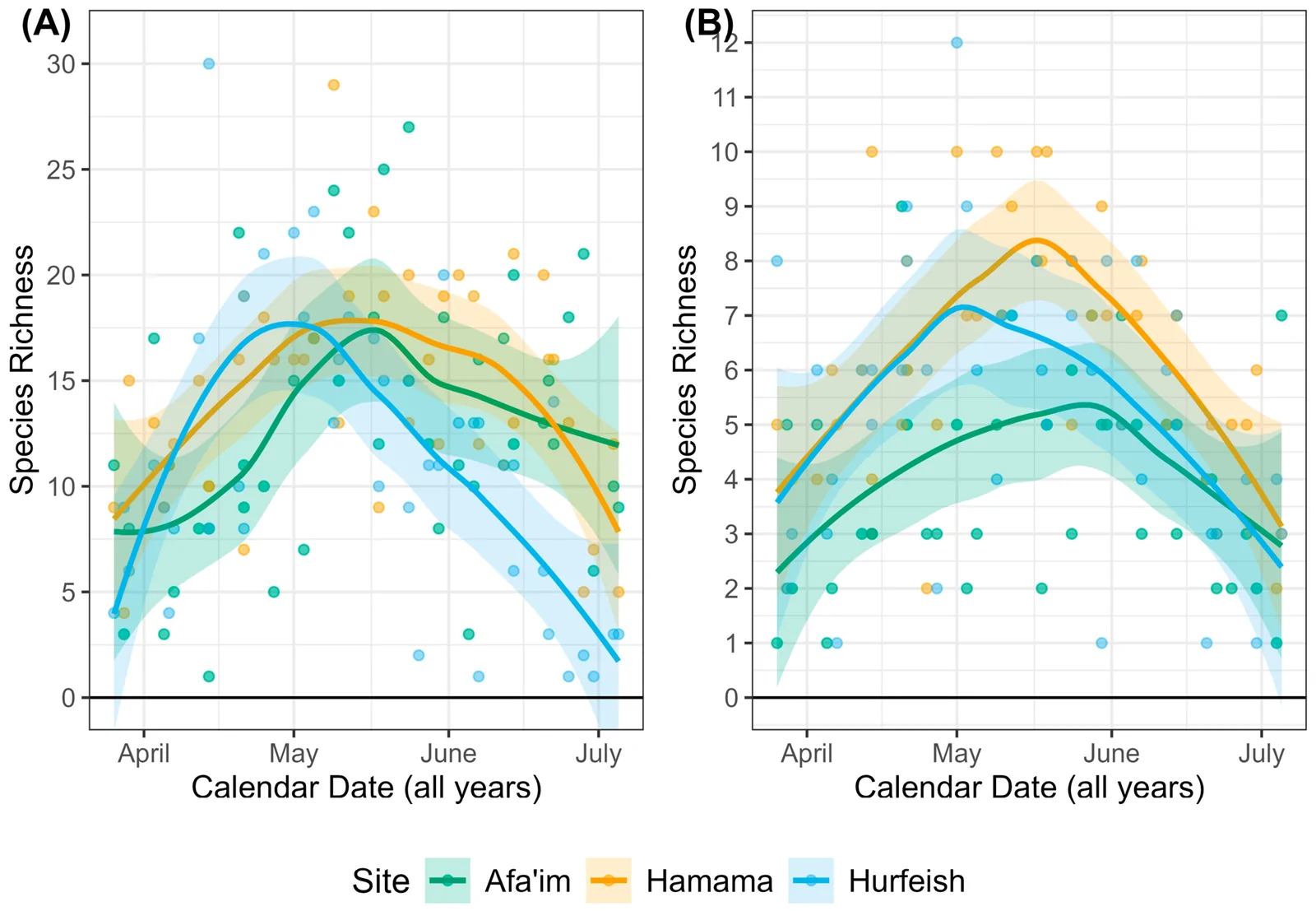
Insect species richness seasonality in Mt. Meron. Points are observations. Lines are LOESS smoothers. (**A**) Beetles. (**B**) Ants. Note the difference in scale between the taxa. Hurfeish peaks in late April; Hamama in early May; Afa’im in mid- May.

The year was dropped from the beetle species richness per survey (site-date combination) model during the model selection process based on AIC, meaning species richness seasonality did not vary significantly between the study years (analysis 4 in Section 2.4). Beetle species richness seasonality is depicted in Figure 3A. Temporal autocorrelation was insignificant in this analysis (SW = 2.013, *p*-value = 0.965). Taken together with the low overlap in species lists between years (Table 1) and the ordinations (Figure 2), this means that while the number of species per survey did not vary considerably between years, the species identities themselves did.

**Figure 4:**
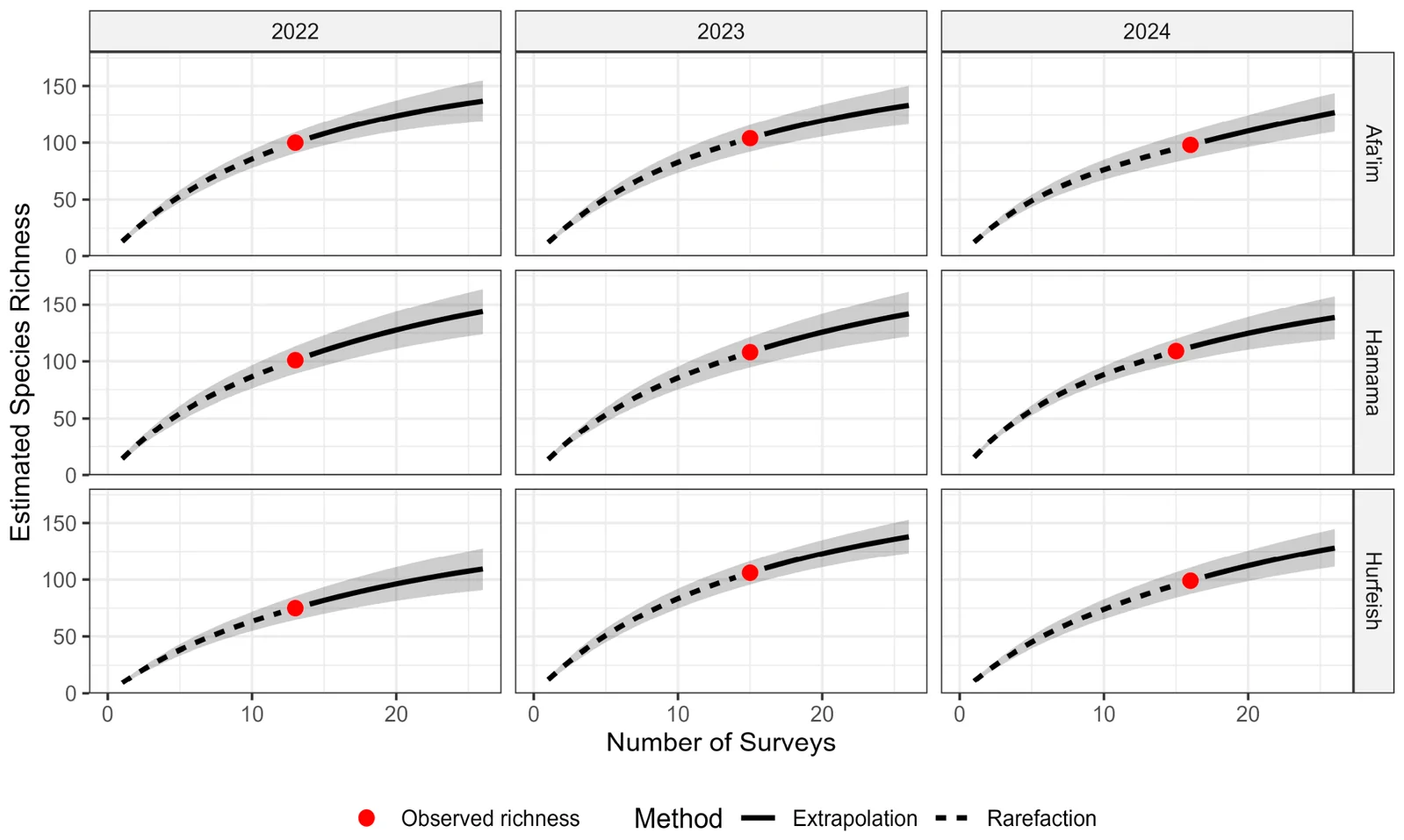
Beetle species accumulation curve by the number of surveys, divided by sites (rows) and years (columns). Red dots are the observed number of species at the actual number of surveys. Lines are the estimated number of species at lower (by rarefaction) or higher (by extrapolation) numbers of surveys. See Section 2.4 for computational details.

**Figure 5:**
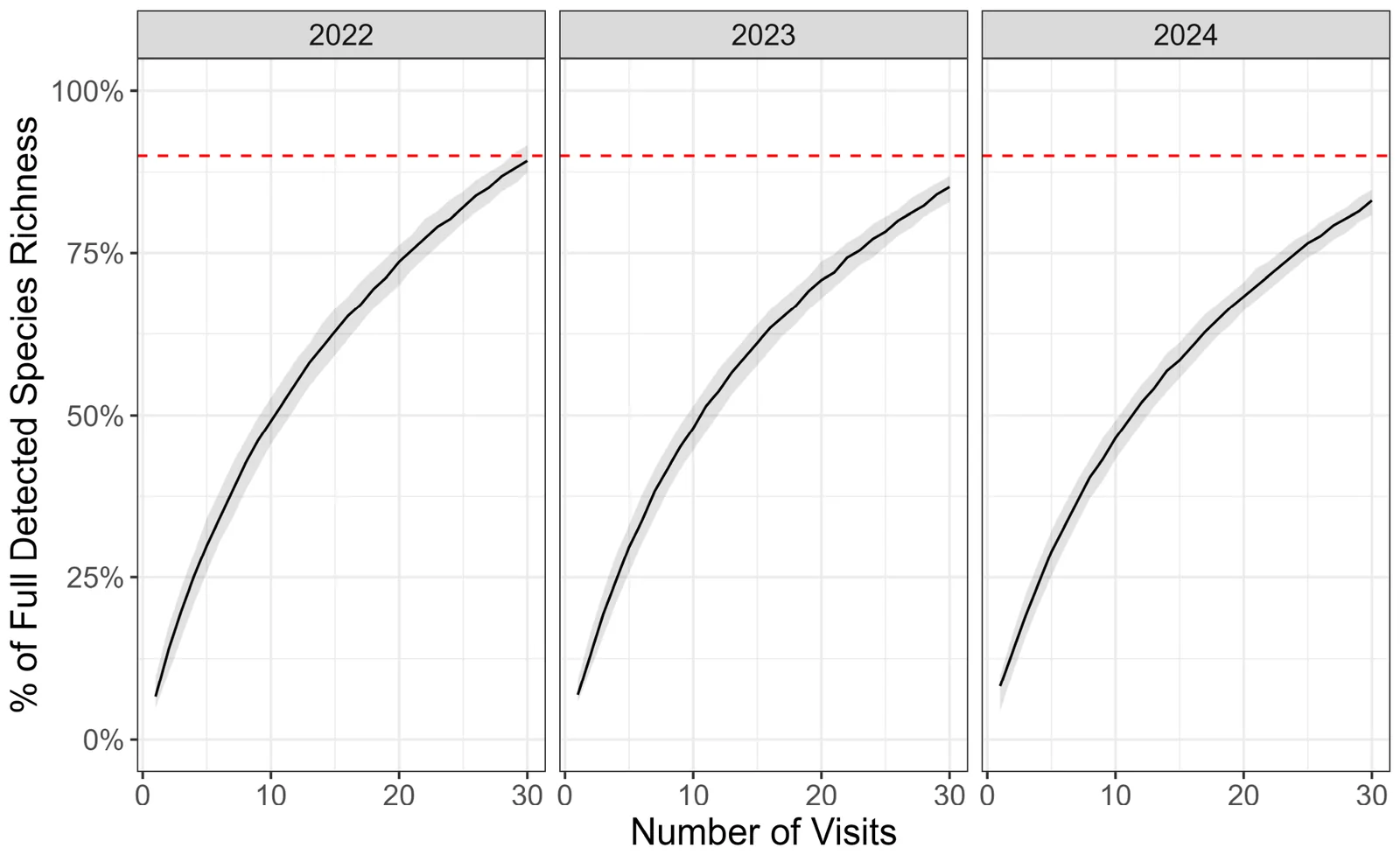
Beetle species accumulation per year by increasing number of visits to each of the three study years. Samples of the possible combinations of taking K visits out of N total visits per year are ordered on the horizontal axis. The percent of the full species richness observed per year was calculated per visit combination sampled and plotted on the y axis. The dashed red line represents 90% of the full species list per year. The line black is the median. The grey ribbon is the inter-quartile range (between the 25th and 75th percentiles).

Analysis of beetle species accumulation as the number of collection visits increases (analysis 5 in Section 2.4) for each of the three study years shows that a “quasi-saturation” of 90% of the total number of species is reached only after ~12 collections per year out of a total of 14 annual visits (Figure 4), or 30 collections across the three years (Figure 5). A formal analysis of asymptotic beetle species richness (analysis 6 in Section 2.4) found that overall (all visits to all three sites throughout the study period) sample coverage was 93.4%, with an estimate of 439.3 species (95% confidence interval: 383.4 to 495.1 species). However, sample coverage was considerably lower for specific site-year combinations, ranging from 62.6% to 77.4%. See Appendix C for further details.

#### 3.1.2. Inter-Site Differences

Table 3 summarizes the species overlap analysis between years (analysis 1 in Section 2.4). Overall, around half of the beetle species appeared only in one site out of three, a quarter to a third in 2/3 sites, and up to a quarter in 3/3 (Table 3). This is similar to the overall phenomenon (total in three years).

Analyzing the seven most common families shows differences, with Melyridae, Apionidae, and Coccinellidae, in which most species are common (appear in three sites), and Chrysomelidae and Curculionidae as the least spatially common ones (Table 4).

The chosen beetle GLLVM (analysis 3 in Section 2.4) found 39 species that were significantly more common in Hamama compared to Afa’im, while 27 species were significantly more common in Afa’im compared to Hamama. The model also found 21 species that were significantly more common in Hurfeish compared to Afa’im, while there were 49 species that were more common in Afa’im compared to Hurfeish. The model found that 23 beetle species were significantly more common in Hurfeish compared to Hamama, while 49 beetle species were significantly more common in Hamama compared to Hurfeish. The full list of species that significantly differed between sites is given in Appendix B.

Species richness (analysis 4 in Section 2.4) was significantly higher in Hamama compared to Hurfeish (*p*-value = 0.0132), but no other significant differences in species richness were found between the sites (Afa’im-Hamama *p*-value = 0.1927; Afa’im-Hurfeish *p*-value = 0.1763).

**Figure 6:**
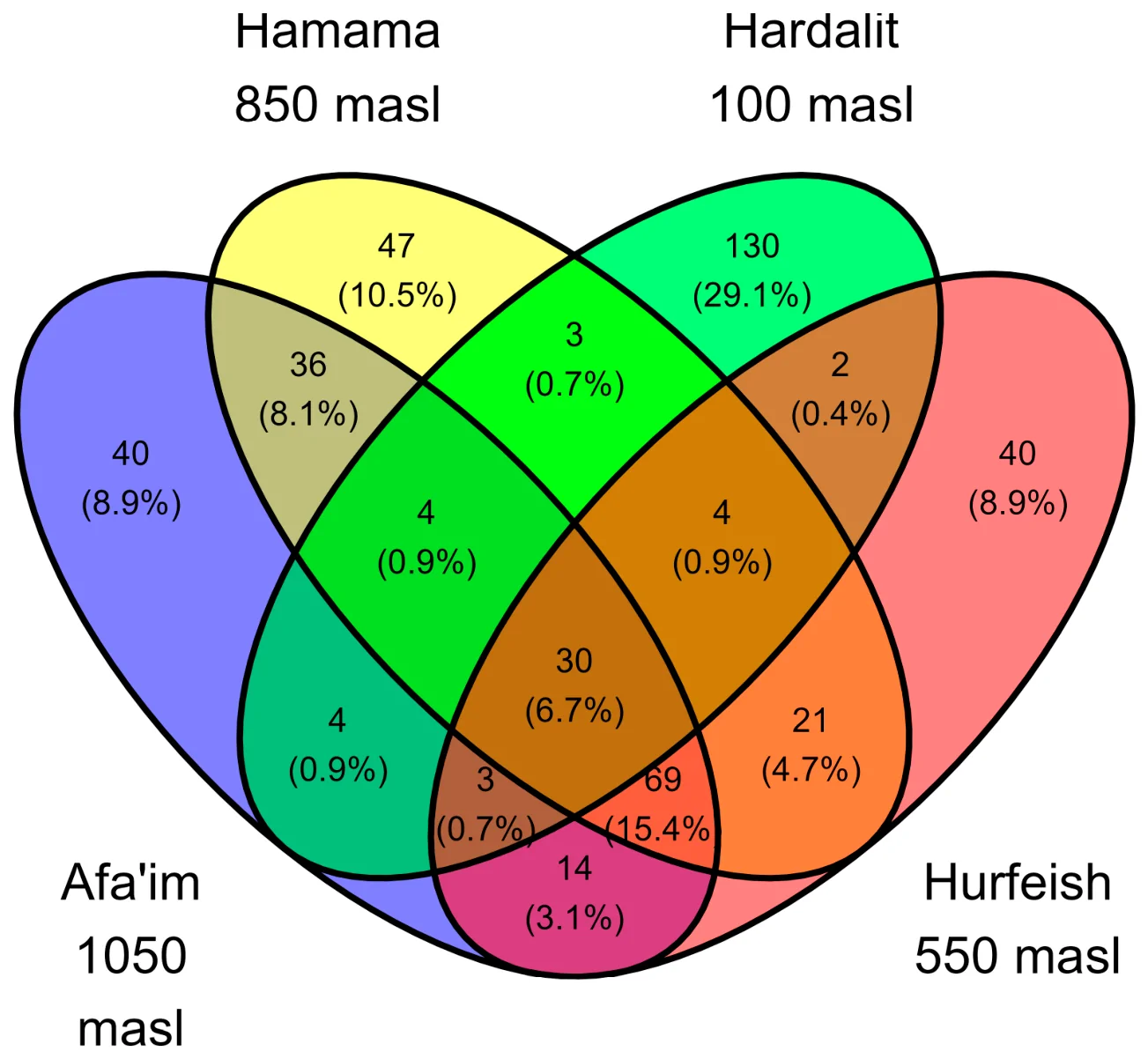
Inter-site differences in beetle species collected at Mt. Meron study sites and at Hardalit site (Row 4, data from [24]).

We compared the three Mt. Meron research sites and the Hardalit site located 20 km downstream and 100 m a.s.l. (Row 4, two-year collection 2020–2021, data from [24]), with limitations resulting from differences in collection years (2020–2021). Figure 6 shows that species unique to one site out of four comprise 57.4% of the total number of observed species; species found in 2/4 of the sites comprise 17.9% of the total number of observed species; species found in 3/4 sites comprise 18% of the total number of observed species; and species found in all four sites comprise 6.7% of the total number of observed species. It is noted that the three sites in Mt. Meron are linked to the Hardalit site via Nahal (stream) Keziv Nature Reserve, creating an ecological corridor along more than 30 km.

**Figure 7:**
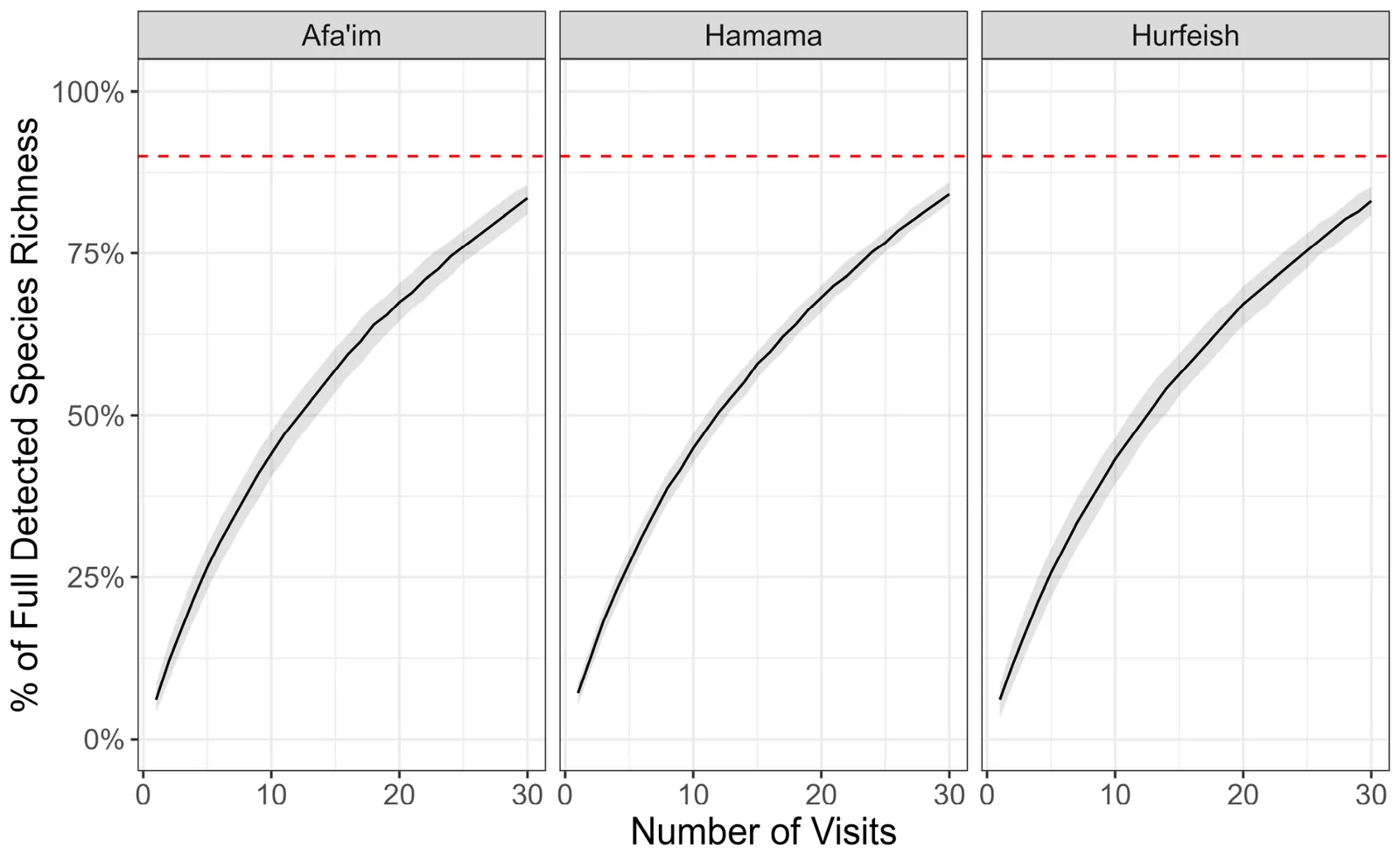
Beetle species accumulation per location by increasing number of visits to each of the three study sites. Samples of the possible combinations of taking K visits out of N total visits per site are ordered on the horizontal axis. The percent of the full species richness observed per site was calculated per visit combination sampled and plotted on the y axis. The dashed red line represents 90% of the full species list per site. The line black is the median. The grey ribbon is the inter-quartile range (between the 25th and 75th percentiles).

Analysis of beetle species accumulation by increasing number of collection visits (analysis 5 in Section 2.4) to each of the three study sites shows that a “quasi-saturation” of 90% of the full observed species list over the entire research period is reached only after over 30 collections in each of the sites (Figure 7).

### 3.2. Ants

#### 3.2.1. Inter-Annual Differences

Nineteen species (38% of the total number of ant species) appeared in all three years, 12 (24%) in 2/3 years, and 19 (38%) only in one year of the three (analysis 1 in Section 2.4). The total number of species was similar across the three years (2022: 31, 2023: 33, and 2024: 36).

Ant species composition per year-site (analysis 2 in Section 2.4) tended to be aggregated by year more than by site (Figure 2B), but this pattern was not as apparent as in the beetles (Figure 2A).

The chosen ant GLLVM for the date-site data (analysis 3 in Section 2.4) found six ant species that were significantly more common in 2023 compared to 2022, while six species were significantly more common in 2022 vs. 2023; six ant species were significantly more common in 2024 compared to 2022, while four species were significantly more common in 2022 vs. 2024. Three ant species were significantly more common in 2024 compared to 2023, while three other species were significantly more common in 2023 vs. 2024. The full list of species that significantly differed between years is given in Appendix B.

Similarly to the beetles, the year was dropped from the ant species richness model during the model selection process following AIC (analysis 4 in Section 2.4). The temporal autocorrelation was insignificant for this analysis as well (DW = 1.787, *p*-value = 0.480). Ant species richness seasonality is depicted in Figure 3B.

**Figure 8:**
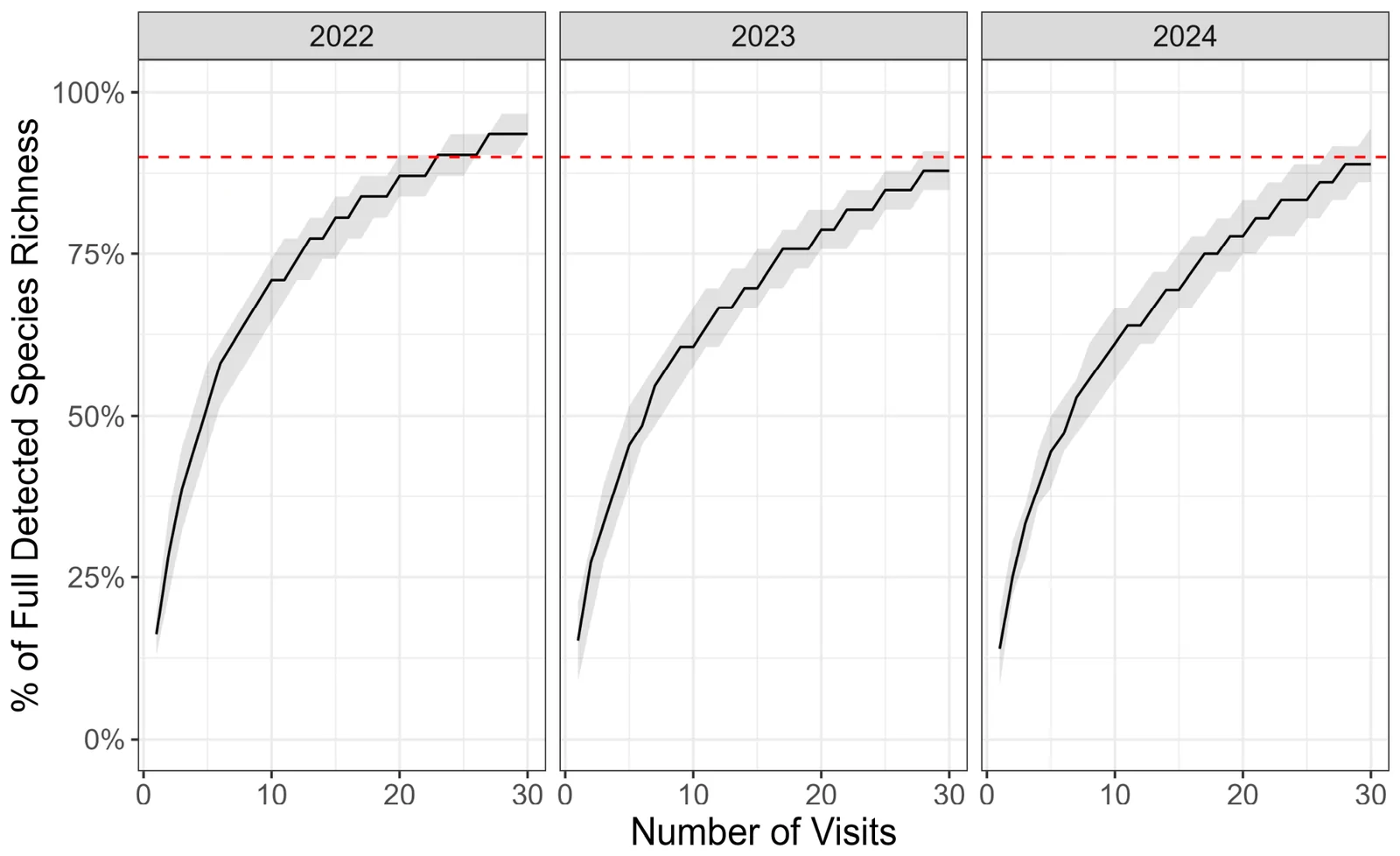
Ant species accumulation per year by increasing number of visits to each of the three study years. Samples of the possible combinations of taking K visits out of N total visits per year are ordered on the horizontal axis. The percent of the full species richness observed per year was calculated per visit combination sampled and plotted on the y axis. The dashed red line represents 90% of the full species list per year. The line black is the median. The grey ribbon is the inter-quartile range (between the 25th and 75th percentiles).

Ant species accumulation per year with increasing number of collection visits (three sites combined; analysis 5 in Section 2.4) shows that 90% of the total number of observed species was reached between ~25 and 30 visits (Figure 8; analysis 5 in Section 2.4). A formal analysis of asymptotic ant species richness (analysis 6 in Section 2.4) found that annual sample coverage varied between 95.4% and 96.1%. See Appendix C for the full details.

#### 3.2.2. Inter-Site Differences

Twenty-four species (48% of the total number of ant species) appeared in three sites, 10 (20%) in 2/3 sites, and 16 (32%) only in one of the three sites (analysis 1 in Section 2.4). The total number of species was relatively similar across the three sites (Hurfeish: 38; Hamama: 38; Afa’im: 32). Although difficult to compare due to sampling differences, in Hardalit (during 2020–2021), a total of 28 species were found in Row 4 (valley bottom), and 47 across the whole site, including those inhabiting the Eastern Mediterranean forest, which is very different from the maquis.

Figure 2B depicts the model-based ordinations of ant species composition by year and site (analysis 2 in Section 2.4). As stated above, inter-site differences were less pronounced than inter-annual differences.

The chosen ant GLLVM (analysis 3 in Section 2.4) found seven species that were significantly more common in Hamama compared to Afa’im, while six other species were significantly more common in Afa’im compared to Hamama. The model also found six species that were significantly more common in Hurfeish compared to Afa’im, while there were eight species that were more common in Afa’im compared to Hurfeish. The model found that four ant species were significantly more common in Hurfeish compared to Hamama, while eight ant species were significantly more common in Hamama compared to Hurfeish. The full list of species that significantly differed between sites is given in Appendix B.

The seasonal peak of ant species richness (analysis 4 in Section 2.4) follows altitude: Hurfeish (550 m a.s.l.) in late April; Hamama (850 a.s.l.) in early May; Afa’im (1050 m a.s.l.) in mid-May (Figure 3B).

**Figure 9:**
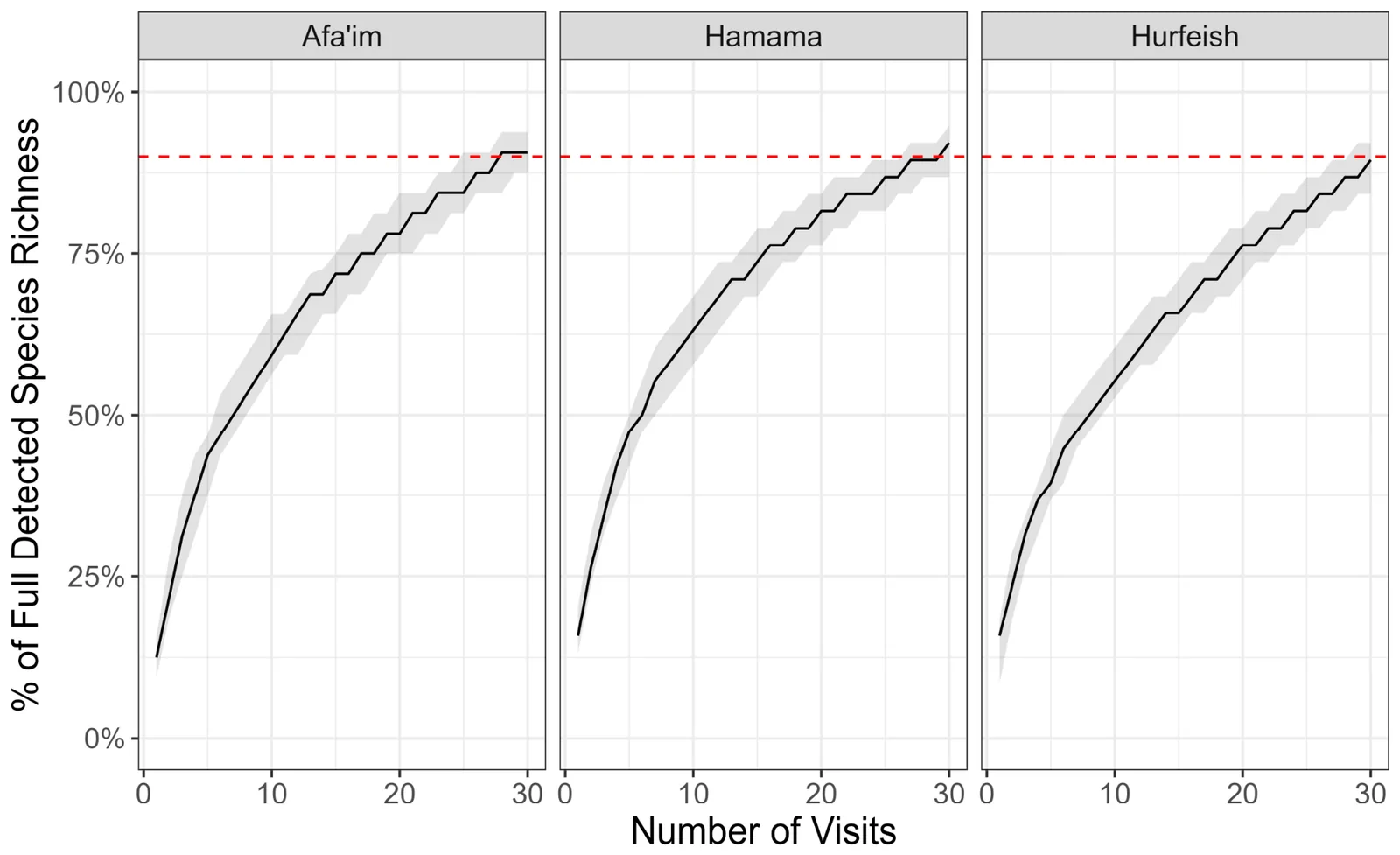
Ant species accumulation per location by increasing number of visits to each of the three study sites. Samples of the possible combinations of taking K visits out of N total visits per site are ordered on the horizontal axis. The percent of the full species richness observed per site was calculated per visit combination sampled and plotted on the y axis. The dashed red line represents 90% of the full species list per site. The line black is the median. The grey ribbon is the inter-quartile range (between the 25th and 75th percentiles).

**Figure 10:**
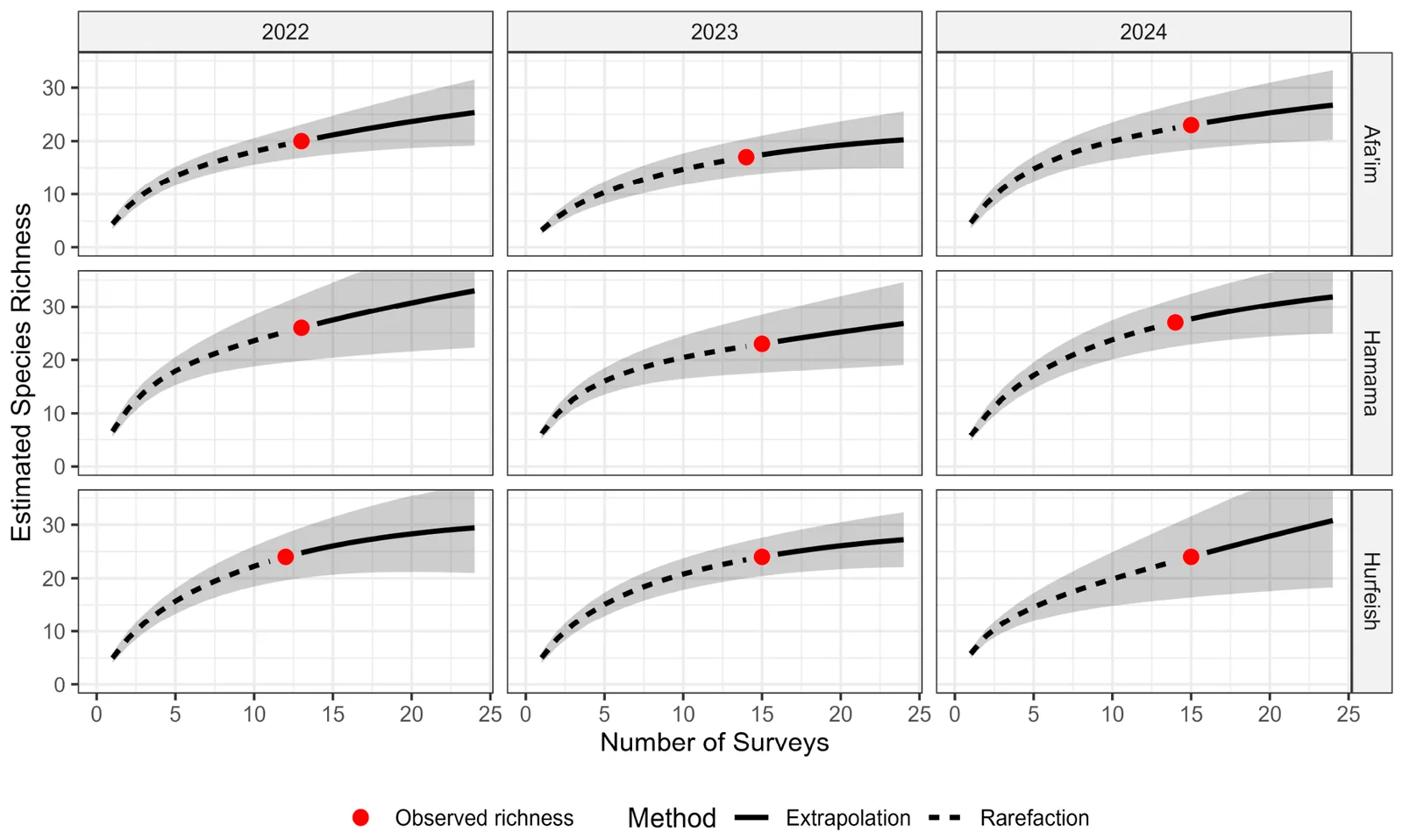
Ant-estimated species richness per site and year, by number of surveys. Red dots are the observed number of species at the actual number of surveys. Lines are the estimated number of species at lower (by rarefaction) or higher (by extrapolation) numbers of surveys. See Section 2.4 for computational details.

Analysis of ant species accumulation with increasing numbers of collection visits (analysis 5 in Section 2.4) at each of the three study sites shows that a “quasi-saturation” of 90% of the total number of species is reached with ~30 collections across the three sites (Figure 9). The asymptotic ant species richness curves (analysis 6 in Section 2.4) demonstrate that species accumulation rate often slows considerably after ~10 annual visits per site (Figure 10).

A formal analysis of asymptotic ant species richness (analysis 6 in Section 2.4) found that overall (all visits to all three sites throughout the study period) sample coverage was 97.9%, with an estimate of 67.2 species (95% confidence interval: 37.1 to 97.3 species). However, sample coverage was considerably lower for specific site-year combinations, ranging from 84.1% to 92.5%. See Appendix C for further details.

## 4. Discussion

### 4.1. Baseline for Insect Biodiversity Monitoring

Our findings present the high biodiversity of flying beetles and ants in Mediterranean mountains and highlands on the one hand, and the difficulty of properly monitoring it on the other hand. Given hundreds of beetle species and dozens of ant species, many of them unique only to a single site or year (Table 1, Table 2, Table 3 and Table 4), along with the inherent limitation of the sweeping method to detected/undetected data (rather than abundance), we demonstrated the considerable surveying effort required to reach a representative species list (Figure 4, Figure 5, Figure 7, Figure 8, Figure 9 and Figure 10) as a baseline for comparison with future surveys, as required in long-term monitoring. This presents a challenge in biodiversity monitoring and conservation, a field with limited resources to begin with, and warrants a discussion on its priorities.

We have shown that limited flying beetle and ant biodiversity monitoring efforts, at least in the Eastern Mediterranean, cannot reliably produce a comprehensive species list, not even for a single site or year, due to very high variation in species detection patterns. Our case exemplifies Didham et al.’s [45] possible “false baseline” and “snapshot” effects—due to a very high level of beetle inter-season temporality within the Eastern Mediterranean spring (Figure 3A), “standard” collection methods of limited collection time per season (examples: four days every season [46]; ten days every season [47]; 5 days in March (dry season) and October (wet season) of each year [48]) may be insufficient to obtain a representative detected species list. Fewer than weekly collections of spring-active flying beetles sampled by sweeping in this region would create both baseline and trend assessment errors. We also warn against Didham et al.’s [45] “popcorn effect”—the potential problem here is validating the extent to which a small set of well-measured local trend estimates can be extrapolated to trends in regional abundance patterns. This challenge is exemplified by the substantial differences among the sites and the essential need to collect from as many sites as possible within the Eastern Mediterranean maquis. While our study is limited in scope to a few sites in a relatively small region, the high inter-annual and inter-site variation we found should serve as a warning for similar studies elsewhere. We suggest that such a pattern might be found in other regions, which would raise similar concerns.

Therefore, without upscaling monitoring efforts (commonly restricted to a few field visits per year over a few sites) and funding, which is unlikely, one cannot reasonably hope to use apparent changes in species lists through time as a reliable indicator for insect biodiversity. Such upscaling is also required for proper occupancy and detectability modeling, which would need large enough sample sizes to account for environmental differences between sites and years. Our results have clearly demonstrated that year-to-year differences in insect biodiversity (especially for highly diverse taxa such as beetles) within the same site are large enough to make the detected species list of any one arbitrary year irrelevant as a baseline, especially given that even 12–14 weekly visits over 3 months yielded relatively low sample completeness assessments (Appendix C). It is thus imperative that researchers find alternative indices or field methods with better reproducibility, i.e., that repeated surveys in similar sites, seasons, and years would produce similar results.

One potential index is species richness per visit. Our results have shown that while species lists varied even between following visits to the same site, species richness remained relatively stable (Figure 3). In our study sites, beetle species richness peaked around May in all three sites, marking this month as the most suitable for a focused insect monitoring scheme. The reliance on species richness on seasonal peak dates should account for both annual weather variations (e.g., certain years might be warmer or rainier than others, which might affect the actual peak season) and long-term climate change. We warn against Didham et al.’s [45] “groundhog effect”—sticking to annual monitoring based on specific dates can lead to mistakes resulting from a phenological shift in insect activity in response to changing climate (winter in Israel is delayed and more intensive than a few decades ago [49]). Another limitation of relying on species richness per visit is that such an index cannot detect species turnover, which could potentially mask the replacement of specialist species with generalist ones. Furthermore, whenever analyzing date-specific biodiversity indices (unlike aggregations such as annual species lists under a given survey effort), weather should be accounted for—an unlucky survey on cloudy or windy days can bias the results.

We therefore recommend repeated visits both before and after the presumed peak season. The peak season could be calculated per year out of a relatively narrow time slot (i.e., shorter than 13–16 weeks, as was done here), and the peak species richness could be compared over the years.

Another optional biodiversity index for a focused monitoring scheme is abundance, both per species (especially for the more common species) and total abundance over all species. As mentioned before, abundance could not be reliably inferred from the sweeping method, and collecting all caught specimens may result in local extinction. However, ants and arboreal beetles in maquis environments collected mainly on flowering shrubs, annuals, and lower branches of trees can also be surveyed using beating sheets [50], whose data can be used to infer abundance and not only detection/non-detection. Other insect survey methods are not as suitable for arboreal or pollinating insects in maquis: pitfall surveys target ground-dwelling taxa, Malaise traps are complicated to use since their results can depend on exact placement and micro-habitat specifications, and pan traps should be set in open areas to be effective. While surveying insects over a focused field season (about 6–8 weeks per year in our region) using beating sheets, researchers can assess not only per-visit species lists but also visit-specific overall abundance and species-specific abundance. This way, the researchers can identify common species in their study system and analyze community composition based on abundance, while omitting rare species.

All of the above suggestions come with the obvious drawback of giving up on finding a comprehensive species list and, as a result, monitoring all species or the full community composition. Researchers should thus frame their conclusions cautiously, by acknowledging their limitations. For example, a study that finds little or no change in species composition based on data collected during a narrow field season cannot reliably claim that the overall fauna changed very little; rather, it can only make claims about species that were sufficiently abundant during that exact narrow field season. In particular, the Eastern Mediterranean beetle fauna has already been found to include many dominant ground-dwelling beetle species active mainly in winter [24].

Our study provides an initial foundation for a future flying insect monitoring scheme in our region by telling researchers their limitations: what proportion of the (spring-active) fauna can be reasonably monitored, and what proportion would remain out of our reach (at least under current funding and field-methods limitations).

### 4.2. Insects’ High Spatial and Temporal Variation Is the Rule, but Is Notably High in the Eastern Mediterranean Maquis

Our results show a substantial spatial (inter-site) divergence within an overall 10 km and 500 m elevation transect of Mediterranean maquis (and 30 km/950 m elevation if we include the ECII Hardalit site, within the limitations of this comparison presented in Methods), which is regarded as broadly similar regional biota, with respect to tree, shrub, mammal, bird, and reptile communities.

Rahbek et al. [51] claimed that mountains, covering about 25% of Earth’s land, harbor over 85% of its amphibian, bird, and mammal species, particularly in tropical biodiversity hotspots. Many of these species are found exclusively in mountain regions.

The Eastern Mediterranean mountains of the Levant were not included in their analysis, probably due to their limited altitude and overall area. Their insect spatial biodiversity probably lies in other reasons (see below).

In our case, the observed spatial variability is not explained solely by broad elevational vegetation zones, as the whole research area lies within Humboldt’s “evergreen forest” within his depiction of elevational habitat layering [51].

Although comparing our findings with other regions or with research on ground-dwelling beetles is difficult, the following studies attest to the notably high biodiversity of the low-altitude, biotic-homogeneous Eastern Mediterranean maquis compared with other low-altitude mountains worldwide. The first two are from the Mediterranean region, although not necessarily the Eastern Mediterranean. Şenyüz et al. [52] collected dung beetles during one year between 469 and 1810 m a.s.l. in Western Anatolia, Turkey, with shifts between a few habitats. Total species richness remained nearly unchanged up to 1200 m, consistent with our findings, with species composition grouped into low altitude (~400–800 m), medium altitude (~900–1600 m), and 1800 m, with substantial overlap between the first and second groups. This is very different from our case, where there is little overlap among all sites regardless of altitude. Another study on dung beetles was conducted over nine months across three vegetation habitats, at elevations between 1120 and 2200 m a.s.l. in a mountain range in the southeastern Iberian Peninsula. Diversity was correlated with elevation—low (4–8 species) up to 1600 m and high (10–14 species) at higher altitudes, according to researchers, due to deforestation at lower altitudes [53].

Chrysomelid beetles were collected in three sampling sites with markedly different types of vegetation communities, along 340-550-1100 m a.s.l. gradient in Peregrina Canyon, Tamaulipas, Mexico. Of the 157 species recorded, 34 were distributed across the entire altitudinal gradient (22%), 40 were recorded only at two sites (25%), and 83 were unique to one of the three sites (53%), a pattern explained by the very different habitats [46]. This overlap pattern is similar to ours (Table 1), although in our case all stations were within the same maquis biota.

A year-long study of seven major arthropod taxa was conducted along a mountain transect (300, 500, 700, 900, and 1100 m a.s.l.) within continuous rainforest in north Queensland, Australia. Researchers found a weak effect of elevation on abundance and diversity [47]. A year-long study of flying beetles along two mountain transects (100–1000 m a.s.l.) of wet tropical forests in the same area, with similar vegetation, rainfall and mean annual temperature (3–4 °C difference), showed a lack of strong elevational patterns, explained by the lack of significant changes in climatic variables along the transect [54]. This is very different from our case, where substantial differences between elevations were detected. Wardhaugh et al. [54] concluded that “it is feasible that low-elevation hills and mountains (of little more than 1000 m) in general do not experience a wide enough range of abiotic conditions to promote the evolution of an elevationally stratified beetle fauna.”

The above-mentioned studies exemplify the notable characteristics of divergence in flying beetle fauna within the supposedly homogeneous Eastern Mediterranean maquis. This may be the result of the region’s unique characteristics, at the junction of Africa, Asia and Europe, which create a species-rich biogeographic unit.

For ants, it is more difficult to compare our results to other studies relating to the influence of elevation on species richness, because most present a hump shape peaking at mid-elevation, which is above ours; this depends on the sites: South Africa: 900 to 3000 m a.s.l.; peak at ~1500 m a.s.l. [55]; Spain: 685 to 2286 m a.s.l.; peak at ~1200 m a.s.l. [56]; Colorado, Nevada and Utah sea level to 4000 m a.s.l.; peaks at ~2000 m a.s.l. [57]; a follow-on study in those U.S. states showed that species richness increased linearly with elevation along two transects (up to 2500 m a.s.l.) and peaked at mid-elevation along a third transect [58]. In Georgia, species richness reaches a maximum at elevations of 800–1200 m a.s.l. [59]. An exception is a peak at 650 m a.s.l., with a decline up to 1500 m a.s.l. across three different biotas (Tanzania: [60]). It seems that within the Mediterranean maquis, at elevations of 500–1000 m a.s.l., there is no notable elevation decline.

While our results are novel regarding the Eastern Mediterranean region, they adhere to the basic observation that species richness grows with area size, which is very well known and emphasized through the Species-Area Relationship (SAR), which is used to predict area size (and other characteristics) as a predictor of species extinction ([61] and reference within); example from ground-dwelling beetles in Italy [62]. While our research focused on establishing a baseline for future monitoring rather than on trends, it emphasizes the need to continue conservation efforts—to guard the research sites presented here for future follow-up monitoring, and to utilize our initial effort as a part of Israel’s national insect monitoring plans.

### 4.3. Study Limitations

Our study attempted to find the baseline for monitoring spring-active Eastern Mediterranean flying beetles and ants. However, despite considerable survey effort within each site and study season, we did not achieve a representative baseline for future monitoring. This result stems from several limitations. First, the short observation period, which included only three study years, was insufficient to produce a comprehensive species list even for our study sites (let alone the region), nor to detect stable species that are likely to be found every year. Second, the limited geographical scope (only three sites) prevented us from reliably studying the impact of altitude or accounting for the spatial structure of the data, despite the fact that both are likely contributing factors to the observed insect occupancy pattern. Third, the absence of quantitative counts, which is inevitable given our monitoring methods and taxa, prevented a more in-depth analysis of the seasonality and geographic pattern of species abundance. Within those limitations, the research provides valuable new data on insect biodiversity in this highly important region.

## 5. Conclusions

A three-year intensive collection effort of spring ants and flying beetles across three elevations (550, 850, and 1050 m a.s.l.) within the Eastern Mediterranean maquis of northern Israel revealed extensive variation among sites and years. The observed site-to-site variation (with similar rock and soil, climate, tree, shrub, mammal, and bird communities) was not simply aligned with broad elevational vegetation zones, but the study design does not allow elevation effects to be separated from other site-specific factors. We hypothesize that this spatial biodiversity main driver is the region’s location at the junction of Africa, Asia, and Europe, which creates a species-rich biogeographic unit. This fact may explain the differences between our findings and those of other studies conducted in low-altitude mountain ranges worldwide. However, from a monitoring perspective, both spatial but mainly temporal (inter-year) variations complicate monitoring efforts. Suggestions were made on this issue. To sum up, the Eastern Mediterranean maquis deserves a much broader arthropod monitoring research effort in natural habitats than it gets today, before ongoing climatic and anthropogenic changes further complicate establishment of robust baseline conditions.

## Figures and Tables

**Table 1:** Inter-annual differences between research sites of Mt. Meron.

Site	Common sp. (3 Years)	2/3 Years	Unique to 1 Year	Total sp. Number
Hurfeish	28 (15.3%)	41 (22.4%)	114 (62.3%)	183
Hamama	26 (12.1%)	52 (24.3%)	136 (63.6%)	214
Afa’im	30 (15%)	42 (21%)	128 (64%)	200
all sites combined	68 (21.5%)	72 (22.7%)	177 (55.9%)	317

**Table 2:** Beetle families with 19 species and more—inter-annual difference in three sites of Mt. Meron.

Family	Total spp.	Unique to 1 Year	2/3 Years	Common spp. (3/3 Years)
Apionidae	20	8 (40%)	5 (25.0%)	7 (35.0%)
Buprestidae	44	25 (56.8%)	10 (22.8%)	9 (20.5%)
Cerambycidae	30	14 (46.7%)	8 (26.7%)	8 (26.7%)
Chrysomelidae	81	50 (61.8%)	18 (22.2%)	13 (16.0%)
Coccinellidae	19	8 (42.1%)	8 (42.1%)	3 (15.8%)
Curculionidae	59	34 (57.6%)	12 (20.4%)	13 (22.0%)
Melyridae	32	18 (56.2%)	6 (18.7%)	8 (25.0%)

**Table 3:** Inter-site differences in beetle species detection between research sites of Mt. Meron.

Year	Common sp. (3 Sites)	2/3 Sites	Unique to 1 Site	Total Number of Species
2022	28 (16.8%)	53 (31.8%)	86 (51.6%)	167
2023	48 (27.4%)	47 (26.8%)	80 (45.7%)	175
2024	32 (17.5%)	59 (32.2%)	92 (50.3%)	183
All years	99 (31.2%)	82 (25.9%)	136 (42.9%)	317

**Table 4:** Beetle families with 19 species and more—inter-site differences across three sites of Mt. Meron.

Family	Common sp. (3 Sites)	2/3 Sites	Unique to 1 Site	Total sp. Number
Apionidae	9 (45.0%)	0 (0.0%)	11 (55.0%)	20
Buprestidae	15 (34.1%)	11 (25.1%)	18 (40.9%)	44
Cerambycidae	9 (30.0%)	7 (23.4%)	14 (46.6%	30
Chrysomelidae	17 (21.0%)	28 (34.5%)	36 (44.4%)	81
Coccinellidae	8 (42.1%)	7 (36.9%)	4 (21.1%)	19
Curculionidae	13 (22.0%)	16 (27.2%)	30 (50.8%)	59
Melyridae	19 (59.4%)	3 (9.3%)	10 (31.2%)	32

## Data Availability

Code and Data are available at: https://doi.org/10.5281/zenodo.23464424 (accessed on 19 October 2026).

## Supplementary Materials

The following supporting information can be downloaded at: https://www.mdpi.com/article/10.3390/biology15211945/s1, and Zenodo (https://doi.org/10.5281/zenodo.23464424). File S1: Ants of Meron Third Year 28.7.2024.xlsx—the ant raw data for this paper; File S2: Beetles Meron and Kziv 2022–2024 from Gilad Ben Zvi 01.12.2025.xlsx—the beetle raw data for this paper, as well as a comparable species list from Finkel et al. 2025 [24]; File S3: 00_Mt_Meron_Wrangling.rmd—The Rmarkdown script (R code and accompanying text and explanation) used for pre-processing the data for further analysis; File S4: 01_Mt_Meron_Venn_diagrams.rmd—The Rmarkdown script (R code and accompanying text and explanation) used for generating the Venn diagrams for this paper; File S5: 02_Mt_Meron_ordinations.rmd—The Rmarkdown script (R code and accompanying text and explanation) used for drawing model-based ordinations for this paper; File S6: 03_Mt_Meron_species_specific_analysis.rmd—The Rmarkdown script (R code and accompanying text and explanation) used for analyzing the community for this paper (species-specific responses to year, location and season); File S7: 04_Mt_Meron_Seasonality.rmd—The Rmarkdown script (R code and accompanying text and explanation) used for drawing species-specific presence-absence data, as well as fitting GLMs for survey-specific species richness; File S8: 05_Mt_Meron_Species_list_per_survey_effort.rmd—The Rmarkdown script (R code and accompanying text and explanation) used for analyzing the expected species list when the sampling effort includes only a portion of actual field trips conducted; File S9: Ants number of detections—by year and site.csv—a table listing the number of detections per ant species, aggregated by both site and year. The number of surveys conducted and the percent of surveys where each species was detected are also provided; File S10: Ants number of detections—by year.csv—a table listing the number of detections per ant species, aggregated by year. The number of surveys conducted and the percent of surveys where each species was detected are also provided; File S11: Ants number of detections—by site.csv—a table listing the number of detections per ant species, aggregated by site. The number of surveys conducted and the percent of surveys where each species was detected are also provided; File S12: Ants number of detections—all data.csv—a table listing the number of detections per ant species in the whole study (not subdivided by site or year). The number of surveys conducted and the percent of surveys where each species was detected are also provided; File S13: beetles number of detections—by year and site.csv—a table listing the number of detections per beetle species, aggregated by both site and year. The number of surveys conducted and the percent of surveys where each species was detected are also provided; File S14: beetles number of detections—by year.csv—a table listing the number of detections per beetle species, aggregated by year. The number of surveys conducted and the percent of surveys where each species was detected are also provided; File S15: beetles number of detections—by site.csv—a table listing the number of detections per beetle species, aggregated by site. The number of surveys conducted and the percent of surveys where each species was detected are also provided; File S16: beetles number of detections—all data.csv—a table listing the number of detections per beetle species in the whole study (not subdivided by site or year). The number of surveys conducted and the percent of surveys where each species was detected are also provided.

## Abbreviations

The following abbreviation is used in this manuscript:

a.s.l	above sea level

## Appendix A. Climate and Vegetation in the Study Area

**Table A1:** Climate differences between the research sites (data from Israel Meteorological Service (URL: https://ims.gov.il/he/ClimateAtlas; accessed on 7 October 2026).

Measure	Hurfeish	Hamama	Afa’im
Mean daily average temp. (C) 2006–2020	18	17	15
Mean daily maximum temp (C) 2006–2020	23	21	20
Mean daily minimum temp (C) 2006–2020	12	12	11
Mean humidity (%) 2006–2020	50	50	55
Daily accumulated solar radiation (kw/hour/1 sq m) 1983–2017	5	5	5
Mean annual precipitation 1991–2020	883	883	988

## Appendix B

**Beetle species inter-annual patterns**

2022 vs. 2023

There are 26 beetle species that are significantly more common in 2023 compared to 2022. These are:

1. *Anthaxia dimidiata*
2. *Anthaxia* sp. 5
3. *Anthaxia* sp. 8
4. *Anthaxia* sp. 9
5. *Aplocnemus* sp. 1
6. *Chrysolina americana*
7. *Clanoptilus assimilis*
8. *Cryptocephalus dahdah*
9. *Curculio* sp.
10. *Dasytiscus* sp.
11. *Ischnopterapion cognatum*
12. *Kalcapion semivittatum*
13. *Longitarsus* sp. 1 yellow
14. *Longitarsus* sp. 2
15. *Longitarsus* sp. 4
16. *Malachius viridanus*
17. *Oenopia* sp.
18. *Oulema melanopus*
19. *Pharoscimnus varius*
20. *Phytoecia virgula*
21. *Podagrica malvae*
22. *Polydrusus bellus*
23. *Rhinocyllus conicus*
24. *Rhinusa* sp.
25. *Smaragdina viridana*
26. *Tychius* sp. 2

There are 32 beetle species that are significantly more common in 2022 compared to 2023. These are:

1. *Acmaeodera crinita abigail*
2. *Acmaeodera ottomana*
3. *Adalia decempunctata*
4. *Adalia* sp.
5. *Anthaxia brevis*
6. *Anthaxia diadema*
7. *Anthaxia klessi*
8. *Anthaxia muliebris*
9. *Anthaxia mundula*
10. *Anthaxia togata*
11. *Attelabus nitens*
12. *Blitopertha nigripennis*
13. *Bruchidius lividimanus*
14. *Bruchidius quinqueguttatus*
15. *Bruchidius* sp. 1 (large)
16. *Bruchidius* sp. 12
17. *Bruchidius varipictus*
18. *Cassida palaestina*
19. *Chlorophorus varius*
20. *Curculio* sp. 1
21. *Danacea marginata* or *maculipennis*
22. *Danacea* sp. (yellow, larger)
23. Coccinellidae Gen. sp. 2
24. *Malthinus* sp. 2
25. *Nephus quadrimaculatus*
26. *Paracorymbia benjamini*
27. *Psylliodes* sp. (large blue)
28. *Psylliodes* sp. (small dark)
29. *Rhagonycha femoralis*
30. *Scymnus* sp.
31. *Stictoleptura cordigera*
32. *Troglops* sp. 5

2022 vs. 2024

There are 21 beetle species that are significantly more common in 2024 compared to 2022. These are:

1. *Acmaeodera ottomana*
2. *Adalia* sp.
3. *Anthaxia dimidiata*
4. *Anthaxia muliebris*
5. *Aplocnemus* sp. 1
6. *Bruchidius* sp. 1 (large)
7. *Chrysolina americana*
8. *Clanoptilus assimilis*
9. *Cryptocephalus dahdah*
10. *Danacea* sp. (yellow, larger)
11. *Ischnopterapion cognatum*
12. *Kalcapion semivittatum*
13. *Oulema melanopus*
14. *Phytoecia virgula*
15. *Podagrica malvae*
16. *Polydrusus bellus*
17. *Psylliodes* sp. (large blue)
18. *Psylliodes* sp. (small dark)
19. *Smaragdina viridana*
20. *Stictoleptura cordigera*
21. *Troglops* sp.5

There are 29 beetle species that are significantly more common in 2022 compared to 2024. These are:

1. *Acmaeodera crinita abigail*
2. *Adalia decempunctata*
3. *Anthaxia diadema*
4. *Anthaxia* sp. 5
5. *Anthaxia* sp. 8
6. *Anthaxia* sp. 9
7. *Aplocnemus* sp. 2
8. *Boveycantharis dimidiatus*
9. *Bruchidius* sp. 12
10. *Bruchidius varipictus*
11. *Chilocorus bipustulatus*
12. *Curculio* sp.
13. *Curculio* sp. 1
14. *Danacea* sp.
15. *Dasytiscus hebraicus*
16. *Dasytiscus* sp.
17. *Hypera jucunda*
18. *Hypera nigrirostris*
19. *Longitarsus* sp. 1 yellow
20. *Longitarsus* sp. 2
21. *Longitarsus* sp. 4
22. *Malachius viridanus*
23. *Malthinus* sp. 2
24. *Oenopia* sp.
25. *Pharoscimnus varius*
26. *Rhagonycha femoralis*
27. *Rhinocyllus conicus*
28. *Rhinusa* sp.
29. *Scymnus* sp.

2023 vs. 2024

There are 27 beetle species that are significantly more common in 2024 compared to 2023. These are:

1. *Acmaeodera crinita abigail*
2. *Acmaeodera ottomana*
3. *Adalia* sp.
4. *Anthaxia brevis*
5. *Anthaxia dimidiata*
6. *Anthaxia klessi*
7. *Anthaxia muliebris*
8. *Anthaxia mundula*
9. *Anthaxia togata*
10. *Aspidapion radiolus*
11. *Blitopertha nigripennis*
12. *Bruchidius lividimanus*
13. *Bruchidius quinqueguttatus*
14. *Bruchidius* sp. 1 (large)
15. *Cassida palaestina*
16. *Chlorophorus varius*
17. *Danacea* sp. (yellow, larger)
18. *Dasytes* sp. 2
19. *Coccinellidae en.* sp. 2
20. *Ischnopterapion cognatum*
21. *Nephus quadrimaculatus*
22. *Oxythyrea noemi*
23. *Paracorymbia benjamini*
24. *Psylliodes* sp. (large blue)
25. *Psylliodes* sp. (small dark)
26. *Stictoleptura cordigera*
27. *Troglops* sp. 5

There are 30 beetle species that are significantly more common in 2023 than in 2024. These are:

1. *Adalia decempunctata*
2. *Anthaxia* sp. 5
3. *Anthaxia* sp. 8
4. *Anthaxia* sp. 9
5. *Aplocnemus* sp. 2
6. *Boveycantharis dimidiatus*
7. *Bruchidius* sp. 12
8. *Bruchidius varipictus*
9. *Chilocorus bipustulatus*
10. *Curculio* sp.
11. *Danacea* sp.
12. *Dasytiscus hebraicus*
13. *Dasytiscus* sp.
14. *Hypera jucunda*
15. *Hypera nigrirostris*
16. *Longitarsus* sp. 1 yellow
17. *Longitarsus* sp. 2
18. *Longitarsus* sp. 4
19. *Malachius viridanus*
20. *Malthinus* sp. 2
21. *Oenopia* sp.
22. *Pharoscimnus varius*
23. *Phytoecia virgula*
24. *Podagrica malvae*
25. *Polydrusus bellus*
26. *Rhagonycha femoralis*
27. *Rhinocyllus conicus*
28. *Rhinusa* sp.
29. *Scymnus* sp.
30. *Tychius* sp. 2

**Beetle species inter-site patterns**

Hamama vs. Afa’im

There are 39 beetle species that are significantly more common in Hamama compared to Afa’im. These are:

1. *Acmaeodera crinita abigail*
2. *Acmaeodera ottomana*
3. *Acmaeodera simulans*
4. *Agrilus* sp. 2
5. *Anthaxia brevis*
6. *Anthaxia diadema*
7. *Anthaxia dimidiata*
8. *Anthaxia eugeniae halperini*
9. *Anthaxia kiesenwetteri*
10. *Anthaxia* sp. 8
11. *Anthaxia togata*
12. *Anthaxia violacea*
13. *Anthonomus chevrolati connexus*
14. *Bangasternus orientalis*
15. *Bruchidius* sp. 1 (large)
16. *Bruchidius* sp. 12
17. *Bruchidius steveni*
18. *Chilocorus bipustulatus*
19. *Clanoptilus assimilis*
20. *Danacea* sp. (yellow, larger)
21. *Dasytes* sp. 3
22. *Delius fugax*
23. *Eomesauletes politus*
24. *Exochomus nigromaculatus*
25. Coccinellidae Gen. sp. 2
26. *Larinus ovaliformis*
27. *Malachius coccineus*
28. *Malthinus* sp. 2
29. *Oenopia* sp.
30. *Oryxolaemus scabiosus*
31. *Oulema melanopus*
32. *Phytoecia virgula*
33. *Polydrusus bellus*
34. *Psylliodes* sp. (large blue)
35. *Rhagonycha femoralis*
36. *Rhinocyllus conicus*
37. *Rhinusa* sp.
38. *Smaragdina viridana*
39. *Troglops* sp. 5

There are 27 beetle species that are more commonly found in Afa’im compared to Hamama. These are:

1. *Adalia* sp.
2. *Anthaxia muliebris*
3. *Anthaxia mundula*
4. *Anthaxia* sp. 5
5. *Anthaxia* sp. 9
6. *Attelabus nitens*
7. *Boveycantharis dimidiatus*
8. *Brancoplia vseteckai*
9. *Ceratapion scalptum*
10. *Chlorophorus varius*
11. *Chrysolina americana*
12. *Clytra novempunctata*
13. *Cryptocephalus dahdah*
14. *Curculio* sp.
15. *Danacea* sp.
16. *Hypera jucunda*
17. *Hypera nigrirostris*
18. *Lasiorhynchites praeustus*
19. *Lasiorhynchites syriacus*
20. *Longitarsus* sp. 2
21. *Longitarsus* sp. 4
22. *Malachius viridanus*
23. *Phrissotrichum tubiferum*
24. *Podagrica malvae*
25. *Procallimus distinctipes*
26. *Protapion politum*
27. *Scymnus* sp.

Hurfeish vs. Afa’im

There are 21 beetle species that are significantly more common in Hurfeish compared to Afa’im. These are:

1. *Acmaeodera simulans*
2. *Agrilus* sp. 2
3. *Anthaxia* brevis
4. *Anthaxia eugeniae halperini*
5. *Anthaxia* sp. 8
6. *Anthaxia* sp. 9
7. *Bangasternus orientalis*
8. *Bruchidius* sp. 12
9. *Bruchidius steveni*
10. *Chilocorus bipustulatus*
11. *Clanoptilus assimilis*
12. *Dasytes* sp. 3
13. *Exochomus nigromaculatus*
14. *Larinus ovaliformis*
15. *Malachius coccineus*
16. *Oenopia* sp.
17. *Oryxolaemus scabiosus*
18. *Oulema melanopus*
19. *Rhinusa* sp.
20. *Smaragdina viridana*
21. *Troglops* sp. 5

There are 49 beetle species that are significantly more common in Afa’im compared to Hurfeish. These are:

1. *Acmaeodera crinita abigail*
2. *Acmaeodera ottomana*
3. *Acmaeoderella flavofasciata*
4. *Anthaxia diadema*
5. *Anthaxia dimidiata*
6. *Anthaxia kiesenwetteri*
7. *Anthaxia muliebris*
8. *Anthaxia* sp. 5
9. *Anthaxia togata*
10. *Anthaxia violacea*
11. *Anthonomus chevrolati connexus*
12. *Aspidapion radiolus*
13. *Attelabus nitens*
14. *Blitopertha nigripennis*
15. *Bruchidius* sp. 1 (large)
16. *Bruchidius varipictus*
17. *Cassida palaestina*
18. *Ceratapion scalptum*
19. *Ceutrohynchus* sp.
20. *Chlorophorus varius*
21. *Chrysolina americana*
22. *Cryptocephalus dahdah*
23. *Curculio* sp.
24. *Curculio* sp. 1
25. *Danacea marginata or maculipennis*
26. *Danacea* sp.
27. *Danacea* sp. (yellow, larger)
28. *Dasytiscus* sp.
29. *Dolichosoma lineare*
30. *Eomesauletes politus*
31. Coccinellidae Gen. sp. 2
32. *Hypera jucunda*
33. *Hypera nigrirostris*
34. *Lasiorhynchites syriacus*
35. *Longitarsus* sp. 1 yellow
36. *Longitarsus* sp. 2
37. *Longitarsus* sp. 4
38. *Malachius viridanus*
39. *Malthinus* sp. 2
40. *Paracorymbia benjamini*
41. *Pedostrangalia riccardoi carmelita*
42. *Pharoscimnus varius*
43. *Phytoecia virgula*
44. *Podagrica malvae*
45. *Polydrusus bellus*
46. *Psylliodes* sp. (large blue)
47. *Psylliodes* sp. (small dark)
48. *Rhagonycha femoralis*
49. *Rhinocyllus conicus*

Hurfeish vs. Hamama

There are 23 beetle species that are significantly more common in Hurfeish compared to Hamama. These are:

1. *Acmaeodera simulans*
2. *Adalia* sp.
3. *Agrilus* sp. 2
4. *Anthaxia eugeniae halperini*
5. *Anthaxia mundula*
6. *Anthaxia* sp. 9
7. *Boveycantharis dimidiatus*
8. *Brancoplia vseteckai*
9. *Bruchidius* sp. 12
10. *Chilocorus bipustulatus*
11. *Clytra novempunctata*
12. *Dasytes* sp. 3
13. *Exochomus nigromaculatus*
14. *Lasiorhynchites praeustus*
15. *Oenopia* sp.
16. *Oulema melanopus*
17. *Phrissotrichum tubiferum*
18. *Procallimus distinctipes*
19. *Protapion politum*
20. *Rhinusa* sp.
21. *Scymnus* sp.
22. *Smaragdina viridana*
23. *Troglops* sp. 5

There are 49 beetle species that are significantly more common in Hamama compared to Hurfeish. These are:

1. *Acmaeodera crinita abigail*
2. *Acmaeodera ottomana*
3. *Acmaeoderella flavofasciata*
4. *Anthaxia diadema*
5. *Anthaxia dimidiata*
6. *Anthaxia kiesenwetteri*
7. *Anthaxia muliebris*
8. *Anthaxia* sp. 8
9. *Anthaxia togata*
10. *Anthaxia violacea*
11. *Anthonomus chevrolati connexus*
12. *Aspidapion radiolus*
13. *Attelabus nitens*
14. *Blitopertha nigripennis*
15. *Bruchidius* sp. 1 (large)
16. *Bruchidius varipictus*
17. *Cassida palaestina*
18. *Ceratapion scalptum*
19. *Ceutrohynchus* sp.
20. *Chlorophorus varius*
21. *Chrysolina americana*
22. *Cryptocephalus dahdah*
23. *Curculio* sp.
24. *Curculio* sp. 1
25. *Danacea marginata or maculipennis*
26. *Danacea* sp.
27. *Danacea* sp. (yellow, larger)
28. *Dasytiscus* sp.
29. *Dolichosoma lineare*
30. *Eomesauletes politus*
31. Coccinellidae Gen. sp. 2
32. *Hypera jucunda*
33. *Hypera nigrirostris*
34. *Lasiorhynchites syriacus*
35. *Longitarsus* sp. 1 yellow
36. *Longitarsus* sp. 2
37. *Longitarsus* sp. 4
38. *Malachius viridanus*
39. *Malthinus* sp. 2
40. *Paracorymbia benjamini*
41. *Pedostrangalia riccardoi carmelita*
42. *Pharoscimnus varius*
43. *Phytoecia virgula*
44. *Podagrica malvae*
45. *Polydrusus bellus*
46. *Psylliodes* sp. (large blue)
47. *Psylliodes* sp. (small dark)
48. *Rhagonycha femoralis*
49. *Rhinocyllus conicus*

**Ant species inter-annual patterns**

2022 vs. 2023

There are six ant species that are significantly more common in 2023 compared to 2022. These are:

1. *Camponotus gestroi* Emery
2. *Crematogaster ionia* Forel
3. *Monomorium venustum*
4. *Tapinoma simrothi*
5. *Temnothorax semiruber* (André)
6. *Temnothorax* sp. 10

There are six ant species that are significantly more common in 2022 compared to 2023. These are:

1. *Aphaenogaster schmitzi* Forel
2. *Crematogaster auberti* Emery
3. *Myrmecina graminicola* (Latreille)
4. *Tapinoma israele*
5. *Temnothorax* cf. *tuberum*
6. *Tetramorium judas* Wheeler and Mann

2022 vs. 2024

There are six ant species that are significantly more common in 2024 compared to 2022. These are:

1. *Crematogaster ionia* Forel
2. *Messor semirufus*
3. *Monomorium venustum*
4. *Tapinoma simrothi*
5. *Temnothorax semiruber* (André)
6. *Temnothorax* sp. 10

There are four ant species that are significantly more common in 2022 compared to 2024. These are:

1. *Aphaenogaster schmitzi* Forel
2. *Camponotus festai* Emery
3. *Myrmecina graminicola* (Latreille)
4. *Temnothorax* cf. *tuberum*

2023 vs. 2024

There are three ant species that are significantly more common in 2024 compared to 2023. These are:

1. *Aphaenogaster schmitzi* Forel
2. *Crematogaster auberti* Emery
3. *Messor semirufus*

There are three ant species that are significantly more common in 2023 compared to 2024. These are:

1. *Camponotus festai* Emery
2. *Myrmecina graminicola* (Latreille)
3. *Temnothorax* cf. *tuberum*

**Ant species inter-site patterns**

Hamama vs. Afa’im

There are seven ant species that are significantly more common in Hamama compared to Afa’im. These are:

1. *Camponotus festai* Emery
2. *Crematogaster auberti* Emery
3. *Crematogaster ionia* Forel
4. *Lasius israelicus* Seifert
5. *Monomorium dentigerum* (Roger)
6. *Myrmecina graminicola* (Latreille)
7. *Tetramorium judas* Wheeler and Mann

There are five ant species that are significantly more common in Afa’im compared to Hamama. These are:

1. *Colobopsis truncata* (Spinola)
2. *Temnothorax* cf. *tuberum*
3. *Temnothorax semiruber* (André)
4. *Temnothorax* sp. 11
5. *Temnothorax* sp. 10

Hurfeish vs. Afaim

There are six ant species that are significantly more common in Hurfeish compared to Afa’im. These are:

1. *Colobopsis truncata* (Spinola)
2. *Crematogaster auberti* Emery
3. *Lasius israelicus* Seifert
4. *Monomorium dentigerum* (Roger)
5. *Temnothorax* sp. 10
6. *Tetramorium judas* Wheeler and Mann

There are eight ant species that are significantly more common in Afa’im compared to Hurfeish. These are:

1. *Camponotus festai* Emery
2. *Camponotus gestroi* Emery
3. *Cataglyphis* sp. near *altisquamis* (André)
4. *Crematogaster ionia* Forel
5. *Monomorium venustum*
6. *Myrmecina graminicola* (Latreille)
7. *Temnothorax semiruber* (André)
8. *Temnothorax* sp. 11

Hurfeish vs. Hamama

There are three ant species that are significantly more common in Hurfeish compared to Hamama. These are:

1. *Colobopsis truncata* (Spinola)
2. *Temnothorax* cf. *tuberum*
3. *Temnothorax* sp. 10

There are eight ant species that are significantly more common in Hamama than in Hurfeish. These are:

1. *Camponotus festai* Emery
2. *Camponotus gestroi* Emery
3. *Cataglyphis* sp. near *altisquamis* (André)
4. *Crematogaster ionia* Forel
5. *Monomorium venustum*
6. *Myrmecina graminicola* (Latreille)
7. *Temnothorax semiruber* (André)
8. *Temnothorax* sp 11

## Appendix C

Asymptotic species richness estimates of beetles and ants using methodology from [31].

Beetles

**Table A2:** Beetle species richness estimates, subdivided by year, site, both or fully aggregated from all study sites and years. The “Observed” column is the total number of species de-facto detected in the study under the described survey effort. The estimator is the estimated number of species under sufficient survey efforts (the asymptotic species richness). Est. s.e. is estimator standard error.

Site	Year	Observed	Estimator	Est. s.e.	95% Lower	95% Upper	Sample Coverage
Afa’im	2022	100	162.1	20.6	121.7	202.5	68.5%
Afa’im	2023	104	165.0	20.4	124.9	205.0	71.6%
Afa’im	2024	98	229.7	37.0	157.2	302.2	74.4%
Afa’im	All	200	340.9	25.7	290.5	391.3	83.6%
Hamama	2022	101	188.4	23.9	141.6	235.3	68.9%
Hamama	2023	108	186.9	21.0	145.7	228.2	71.3%
Hamama	2024	109	173.2	21.1	131.9	214.4	77.4%
Hamama	All	214	316.1	22.0	273.0	359.2	84.6%
Hurfeish	2022	75	145.9	25.1	96.7	195.1	62.6%
Hurfeish	2023	106	176.0	18.3	140.17	211.9	69.5%
Hurfeish	2024	99	180.6	27.5	126.7	234.5	68.1%
Hurfeish	All	183	295.9	27.8	241.57	350.3	82.6%
All	2022	167	239.3	18.0	204.0	274.7	85.7%
All	2023	175	250.4	21.1	208.9	291.8	88.4%
All	2024	183	253.5	22.4	209.5	297.4	88.4%
All	All	317	439.3	28.5	383.4	495.1	93.4%

Ants

**Table A3:** Same as Table A2, but for ants instead of beetles.

Site	Year	Observed	Estimator	Est s.e.	95% Lower	95% Upper	Sample Coverage
Afa’im	2022	20	34.8	15.1	5.2	64.3	86.8%
Afa’im	2023	17	22.7	9.0	5.1	40.2	86.0%
Afa’im	2024	23	33.0	11.3	10.8	55.1	89.2%
Afa’im	All	32	35.9	10.0	16.2	55.6	95.6%
Hamama	2022	26	49.1	23.6	2.9	95.3	88.8%
Hamama	2023	23	45.9	18.6	9.3	82.4	92.5%
Hamama	2024	27	36.3	16.4	4.1	68.5	88.4%
Hamama	All	38	57.7	18.3	21.9	93.5	95.8%
Hurfeish	2022	24	31.6	9.7	12.7	50.6	85.0%
Hurfeish	2023	24	30.0	11.2	8.1	51.9	90.3%
Hurfeish	2024	25	109.9	47.8	16.2	203.7	84.1%
Hurfeish	All	39	61.0	16.8	28.0	93.9	93.4%
All	2022	31	62.2	18.6	25.6	98.7	96.1%
All	2023	33	45.2	14.3	17.2	73.3	95.4%
All	2024	37	46.9	11.8	23.8	69.9	95.5%
All	All	51	67.2	15.4	37.1	97.3	97.9%
